# Macrophages in immunoregulation and therapeutics

**DOI:** 10.1038/s41392-023-01452-1

**Published:** 2023-05-22

**Authors:** Shanze Chen, Abdullah F.U.H. Saeed, Quan Liu, Qiong Jiang, Haizhao Xu, Gary Guishan Xiao, Lang Rao, Yanhong Duo

**Affiliations:** 1 Department of Respiratory Diseases and Critic Care Unit, Shenzhen Institute of Respiratory Disease, Shenzhen Key Laboratory of Respiratory Disease, Shenzhen People’s Hospital (The Second Clinical Medical College, Jinan University; The First Affiliated Hospital, Southern University of Science and Technology), Shenzhen, 518020 China; 2 Department of Cancer Biology, Beckman Research Institute of City of Hope National Medical Center, Los Angeles, CA 91010 USA; 3 Department of Laboratory Medicine, Huazhong University of Science and Technology Union Shenzhen Hospital (Nanshan Hospital), Shenzhen University, Shenzhen, 518052 China; 4 Department of Respiratory, The First Affiliated Hospital, School of Medicine, Southern University of Science and Technology, Shenzhen, 518055 China; 5 State Key Laboratory of Fine Chemicals, Department of Pharmaceutical Sciences, School of Chemical Engineering, Dalian University of Technology, Dalian, China; 6 Institute of Biomedical Health Technology and Engineering, Shenzhen Bay Laboratory, Shenzhen, 518132 China; 7 Department of Microbiology, Tumor and Cell Biology (MTC), Karolinska Institutet, Stockholm, Sweden

**Keywords:** Innate immune cells, Tumour immunology

## Abstract

Macrophages exist in various tissues, several body cavities, and around mucosal surfaces and are a vital part of the innate immune system for host defense against many pathogens and cancers. Macrophages possess binary M1/M2 macrophage polarization settings, which perform a central role in an array of immune tasks via intrinsic signal cascades and, therefore, must be precisely regulated. Many crucial questions about macrophage signaling and immune modulation are yet to be uncovered. In addition, the clinical importance of tumor-associated macrophages is becoming more widely recognized as significant progress has been made in understanding their biology. Moreover, they are an integral part of the tumor microenvironment, playing a part in the regulation of a wide variety of processes including angiogenesis, extracellular matrix transformation, cancer cell proliferation, metastasis, immunosuppression, and resistance to chemotherapeutic and checkpoint blockade immunotherapies. Herein, we discuss immune regulation in macrophage polarization and signaling, mechanical stresses and modulation, metabolic signaling pathways, mitochondrial and transcriptional, and epigenetic regulation. Furthermore, we have broadly extended the understanding of macrophages in extracellular traps and the essential roles of autophagy and aging in regulating macrophage functions. Moreover, we discussed recent advances in macrophages-mediated immune regulation of autoimmune diseases and tumorigenesis. Lastly, we discussed targeted macrophage therapy to portray prospective targets for therapeutic strategies in health and diseases.

## Introduction

The primary formation of host resistance, contrary to infectious pathogens in disease, is the innate immune framework mainly made of innate immune cellular entities and cells originating from myeloid, comprising macrophages, monocytes, granulocytes, and dendritic cells (DCs).^1,2^ When disease ensues, pathogen-associated molecular patterns (PAMPs) and damage-associated molecular patterns (DAMPs) are recognized via the cell surface or intracellular pattern recognition receptors (PPRs) from innate immune cells, for example, Toll-like receptors (TLRs), advanced glycation end products (RAGE) and Nod-like receptors (NLRs).^2^ Successively, the interaction of PAMPs or DAMPs through particular receptors triggers signaling cascades following association at reactive oxygenic and nitrogen-related species, chemokines, proinflammatory cytokines, and antimicrobial-associated peptides, including augmented phagocytosis and the efficient elimination of microbial infection.^2,3^ In addition, instigating the innate immune system leads to the follow-up with the trigger of more specific adaptive immunity. However, given the advantages of the innate immune system, this tends to remain a double-edged sword employing inflammation that will damage the host.^2^ Hence, it is significant to comprehend the immune regulatory strategies that govern the inflammatory progression’s commencement, extent, and goals.

Macrophages are innate immune cells first identified by Elia Metchnikoff in starfish hatchlings in 1882 when tangerine tree thistles were used, then in *Daphnia magna* or essential water flea infested with fungal spores as the cells responsible for the cycle of phagocytosis of foreign materials. For this accomplishment, the Nobel Prize (Physiology and Medicine) was presented to Elia Metchnikoff in 1908. In this way, macrophages are the primary innate immune cells identified 130 years ago.^4^

Macrophages are crucial in innate immunity by regulating several homeostatic and evolutionary host defense immune responses. Besides, macrophages partake in many other biological events, including modulating reactive oxygen species (ROS) endogenous intensities, iron homeostasis, tissue injury repair, and numerous other metabolic functions.^5,6^ In addition, macrophages have three essential functions, i.e., immunomodulation, phagocytosis, and antigen presentation. They are vital for performing normal immune reactions under various pathophysiological conditions.^7^

As the relationship between TAMs and malignant tumors expands into more apparent, TAMs are suggested as possible biomarkers for diagnosing and prognosis tumors and therapeutic targets in various cancers. Inhibiting monocyte recruitment, targeting TAM activation, converting TAMs to anticancer macrophages, and targeting TAMs in conjunction with conventional chemotherapy are all examples of the therapeutic strategy aimed at TAMs.^8^

Macrophages accomplish widespread functions counting tissue repair and regulation of homeostasis and immunity. Nevertheless, the signal transduction details are still far from complete. Moreover, even though these cells are precisely regulated, immune pathways modified in disease and health are poorly understood. Hence, we have comprehensively reviewed the essential functions of macrophages in various immune settings that will support an in-depth understanding of recent advances in macrophage signaling and immune regulation.

## Activation and polarization of macrophages

An essential function of macrophages is to sanitize the cellular fragments produced by tissue remodeling and apoptosis, leading to cell death.^9^ For these events, macrophages can sense danger signals over pattern recognition receptors (PRRs), consisting of TLRs and C-type lectin receptors (CLRs). Afterward, the elicited danger-associated signals comprise PAMPs on the attacking entities and DAMPs, which happen as the infection advancements, and cells are destroyed.^2,10^ Bone marrow-derived monocytes are the origins of the macrophages, the primary defense in the front line to entering infectious pathogens, acting as a surveillance framework, and an essential constituent of innate immunity.^11,12^

### Macrophage polarization

**Fig. 1:**
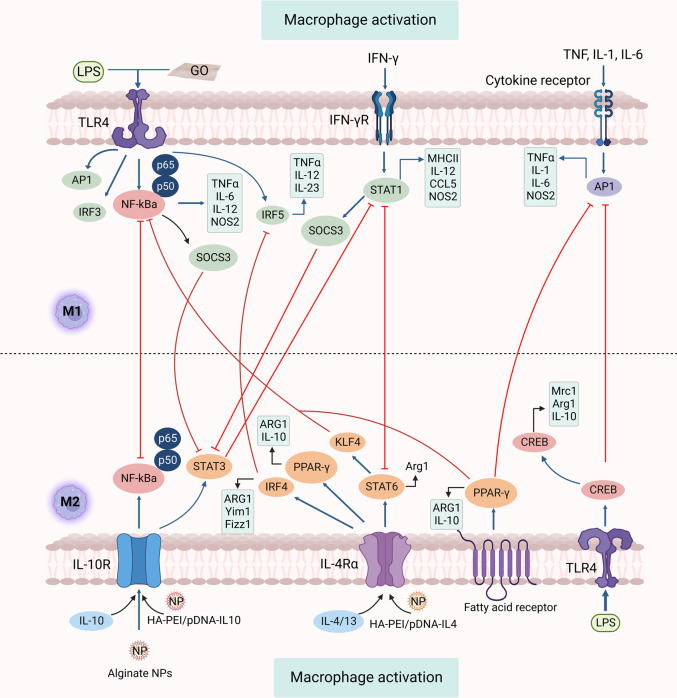
Pathways for signaling macrophage polarization. The figure demonstrates numerous strategies essential for macrophage polarization and depicts feedback control on signaling pathways of M1 and M2. Key signal channels include IRFs, STATs, NF-κB, and SOCS. The downstream protein STAT6 is krüppel-like factor 4 (KLF-4). Also, macrophage polarization can be induced by GO (graphene oxide) towards the M1 phenotype. HA-PEI/pDNA-IL-10 or HA-PEI/pDNA-IL-4 NPs) and tuftsin-modified alginate NPs containing murine cytokine IL-10 plasmid DNA modulate programming from M1 toward M2. Similarly, enhanced expression of API, PPARγ, and CREB is mediated by cytokine receptor, fatty acid receptor, and TLR4, respectively. STAT1-STAT6 introduces the feedback control of M1 and M2, IRF5-IRF4, NF-κB-PPARγ, AP1-CREB, and AP1-PPARγ, which play a crucial role in inflammatory disease instigation, development, and termination. TLR toll-like receptor, CREB cyclic AMP-responsive element binding, NF-κB nuclear factor kappa light chain enhancer of activated B cells, STAT signal transducers and activators of transcription, PPARγ peroxisome proliferator-activated receptor γ, IRF interferon regulatory transcription factor, API apigenin

The macrophages’ destiny relies on various environmental conditions that fuel polarization to any of the classically triggered pro-inflammatory M1 response or triggered M2 immune response. Macrophage M1 or M2 polarization is a precisely regulated process comprising several key signaling pathways, transcriptional epigenetic and post-transcriptional regulatory networks (Fig. 1).^13,14^

Immune-related responses are elicited by several pathogen-associated molecular markers, including damage-associated molecular markers, lipopolysaccharide (LPS), as well as interferon-γ (IFN-γ), a type 1 helper T (Th1) cytokine-provoking pro-inflammatory responses.^2,15^ In addition, Th2 cytokines, such as interleukin (IL)-4 and IL-13, trigger alternative M2 immune reactions. Likewise, diverse factors affect macrophage polarization and reprogramming (Table 1).^2,16^

**Table 1:** Factors influencing macrophage polarity and reprogramming

Factors	M1 macrophage	M2 macrophage	Ref.
Stimulation	LPS, IFN-γ, GM-CSF, TNFα	LPS, ICs, GCs, LIF, adenosine, TGF-β, IFN-γ, IL-4, IL-10, IL-13, IL-33, Th cytokine, M-CSF, helminth	^17^
Cytokines	High TNFα, IL-1β, IL-6, IL-12, IL-18, IL-23	Low IL-12, IL-23, high IL-1RA, IL-10, IL-4/IL-13	
Markers	CD86, CD80, CD68, MHC II, IL-1R, TLR2, TLR4, iNOS, SOCS3	CD86, CD163, MHC II, SR, MMR/CD206, CD200R, TLR1, TLR8, VEGF, MMP9, TGM2, DecoyR, IL-1R, IIMR, Fizz-1, Ym1	
Chemokines	CXCL1, CXCL3, CXCL5, CXCL8, CXCL9, CXCL10, CXCL11, CXCL13, CXCL16 CX3CL1, CXCR3; CCL2, CCL3, CCL4, CCL5, CCL8, CCL11, CCL15, CCL19, CCL20; NOS2, CD64, IDO, SOCS1	CCL1, CCL2, CCL5, CxCL10, CCL13, CCL14, CCL17, CCL18,CCL22, CCL23, CCL24, CCL26, CXCL16, CCR2, CCR3, CCR4	
Reprogramming	Reprogramming from M2 to M1; Superparamagnetic iron-oxide nanoparticle (SPIONs), Glycocalyx-mimicking NPs (glycol-NPs), Ag, Au, Co, ZnO, TiO_2_, SiO_2_	Reprogramming from M1 to M2; hyaluronic acid-poly (ethyleneimine), plasmid DNA NPs (HA-PEI/pDNA), miR-223-expressing plasmid DNA-encapsulated, HA-PEI NPs (HA-PEI/miR-223 NPs), alginate NPs, Au, Ti	
Other macrophage-induced products	STAT1, STAT2, IRF5, p65, iron uptake, and metabolism; high ROS, ROIs, NO, and MHC I, low IL-10	STAT3, STAT6, IRF4, p50, iron release, folate uptake and metabolism, arginase 1 to polyamine	

In comparison, different terminology and definitions contribute to the stimulation and polarization of the macrophage. For example, recent research reported M1 and M2 phenotypes in Balb/c and C57BL/6 mice.^17,18^ In another study, Abdelaziz et al. depicted that IL-4 and IL-13 trigger alternative macrophage activation.^19^ Furthermore, other studies reported a regulatory macrophage (Mreg) activation.^20^ Another analysis was defined as classical IFN-α macrophage stimulation.^21^ Again, oxidized lipids in antioxidant macrophages (Mox) are revealed to induce macrophage phenotype.^22^ In addition, Yi Cai et al. identified a subset of macrophages that are distinguished with high expression of CXCL4 (M4)^23^ by using single-cell transcriptome analysis. Lv et al. described alternate triggered macrophages into M2a, b, c.^24^ Moreover, a new study reveals for the first time that insulin-like growth factor 2 (IGF2) mRNA-binding protein 2 (IGF2BP2) plays an essential role in macrophage activation.^25^

#### Stimulation of M1

Microbial products or pro-inflammatory cytokines induce M1 polarized macrophage (M1). The critical Th1 cells-derived inflammatory mediator that polarizes macrophages to the M1 phenotype is IFN-γ. The binding of IFN-γ to its receptor interferon-gamma receptor (IFNGR) firstly activates the Janus kinase (Jak) adapters, which subsequently leads to the activation of STAT1 (signal transducer and transcription activator 1).^26^ In addition, IFN-α also triggers specific gene expression profiles, including major histocompatibility complex (MHC) II, IL-12, nitric oxide synthase 2 (NOS2), and suppressor of cytokine signaling (SOCS)1. IFN-γ is part of the M1/M2 model paired with LPS. Nevertheless, the combination’s gene expression profiles vary from LPS and IFN-γ alone.^27^

The PRRs, such as TLRs, recognize bacterial moieties. Specifically, LPS and other microbial ligands activate TLR4, activating the TIR-domain-containing adapter-inducing interferon-β (TRIF) and the myeloid differentiation response 88 (MyD88). The TRIF-regulated mechanism triggers kinase cascades and eventually activates the interferon-responsive factor 3 (IRF3). IRF3 tracks the secretion of IFNs, including IFN-α and IFN-β. In TLR4, MyD88, which is yet another adapter, activates the nuclear factor kappa-B (NF-kB) pathway (p65 and p50), a crucial transcription factor in the polarization of M1 macrophages. MyD88 also activates the protein activator 1 (AP-1) via MAPK.^28,29^ These pathways promote the expression of various inflammatory genes like proinflammatory cytokines (including tumor necrosis factors (TNF), IL-1β, and IL-12), chemokines CXCL10, CXCL11, co-stimulating proteins, and proteins that process antigens.^30^

#### Stimulation of M2

Cytokine IL-4 and IL-13 bind to the IL-4Rα receptor and M2 polarization. Generally, JAK1 and JAK3 signals activate STAT6, which translocates into the nucleus and modulates interferon genes-mediated antiviral innate immune responses.^31^ Other transcription factors involved IRF4 and peroxisome proliferator-activated receptor γ (PPARγ). A variety of proteins, such as arginase 1 (Arg 1), Ym1 (or Chitinase 3-like 3, Chi3l3), resistin-like-α (Retnla or Fizz1), CCL17 and CD206 (or macrophage mannose receptor 1, Mrc1) are controlled by STAT6, IRF4, and PPARγ. The PPARγ transcription factor can also be triggered by fatty acid receptors linking free fatty acids.^19^

All the leukocytes may contain IL-10, which binds the heterodimers IL-10 (IL10-R1 and IL10-R2). The IL-10 binds to IL-10R leading to receptor autophosphorylation, which activates the STAT3 transcription factor. The binding of STAT3 to its promotor can modulate the expression of the suppressor of cytokine signaling 3 (SOCS3), which blocks the proinflammatory cytokine signaling pathways.^32^

As a product of digestion by cellular enzymes in macrophages, glucocorticoids are formed from glucocorticoid hormones. They will pass across the membrane and are lipophilic. The glucocorticoid receptor (GR) binds with intracellular glucocorticoids, resulting in the complex’s nuclear translocalization.^33^ The DNA is directly linked to the complex, and anti-inflammatory genes like IL-10 and IL1-R2 are prompted to transcribe. Conversely, the GR complex can communicate with other transcription factors, such as NF-kB or AP-1.^34,35^

#### Attributes of macrophage polarization

The concept of M1/M2 polarization was reported in 2000 based on the capacity of C57BL/6J macrophages to generate NO (M1 polarized) compared to Balb/c mice (M2 polarized).^36,37^ Altered LPS infusion metabolic activity of arginine evokes various macrophage-related phenotypes in C57BL/6J mice and albino mice in Balb/c. C57BL/6J peritoneal macrophages can be induced to express inducible NOS (iNOS), generating NO and Th1 CD4^+^ T cells mediated immune response. In comparison, Balb/c albino mice added ornithine-like arginase and a Th2-like response system. The macrophages called M1 and M2 looked the same as Th1 and Th2.^37–40^ Such findings suggested that macrophages urging various mouse species had an alternative preference attributable to LPS to produce NO and arginase.

In addition, NO formation is a fundamental feature of M1 macrophages following the upregulation of the inducible NOS2.^41^ Large quantities of proinflammatory cytokines and ROS, comprising tumor necrosis factor-alpha (TNF-α), IL-1β, IL-12, and IL-23, are auxiliary generated by these classically activated M1 macrophages, adding the killings of pathogens and recruitment of additional proinflammatory cell sorts.^42,43^ Graphene oxide (GO) can aid in forming an antioxidant and reducing inflammation and inflammatory macrophage polarization through lessening ROS in the cell.^44,45^ GO is a critical transporter for IL-4 plasmid DNA (IL-4 pDNA) that proliferates M2 macrophages.^44,45^ Conversely, M2 macrophages participate in tissue remodeling, wound restoration, regulation of tumor environment, hypersensitive reactions, and responses to helminths.^46,47^ These, on the other hand, actuated macrophages with improved arginase action and IL-10 production. Because of the widespread assorted variety of M2 macrophage roles, they can further be subdivided into Mregs based on the alternatively activated subset, myeloid-derived suppressor cells (MDSCs), profibrotic macrophages (M2a), and TAMs.^48^ Respectively, the M2 subtypes are generally immunosuppressive but have discrete functions of activators and effectors. Mregs are mutually stimulated, produce IL-10, and induce neither arginase nor NOS2, nonetheless playing their role in repressing M1 macrophages.^49^ The tumor factors induce the isolation and polarization of TAMs present in tumor microenvironments (TME) (e.g., hypoxia) and typical M2 stimuli. This term triggers tumor growth to be instigated and encouraged similarly through immune suppression and angiogenesis.^50^ MDSCs are believed to be a precursor of TAMs. Nevertheless, as seen in mice, they also increased the supply of GR1, a proinflammatory immune marker, decreased the activation of F4/80, and had a corresponding role in arginase and NOS2. MDSCs play a crucial role in inhibiting innate and T-cell responses in cancer. M2a macrophages produce fibronectin and other IL-4 and IL-13, enhancing the repair of injuries and the development of extracellular matrix (ECM).^50^ After activation, macrophages maintain plasticity and shift from one functional phenotype to a different one centered on conditions. Nevertheless, extreme response of any polarization state can influence tumor formation, tissue necrosis, inflammation, and fibrosis.^51^ Anti-programmed death-ligand 1 (PD-L1) therapy in colorectal cancer (CRC) may be achieved by optimizing the release of sEV- microRNAs (miRNAs) from CRC and addressing PD-L1 in TAMs, according to recent research.^52^ In a breast cancer model, PYK2 controls TAMs. There is an indication that PYK2 depletion alone in macrophages significantly lowers the amount of TAMs and slows tumor development and angiogenesis.^53^ Hence, it is substantial to comprehend the immune approaches of macrophage regulation for therapy and disease management.

**Fig. 2:**
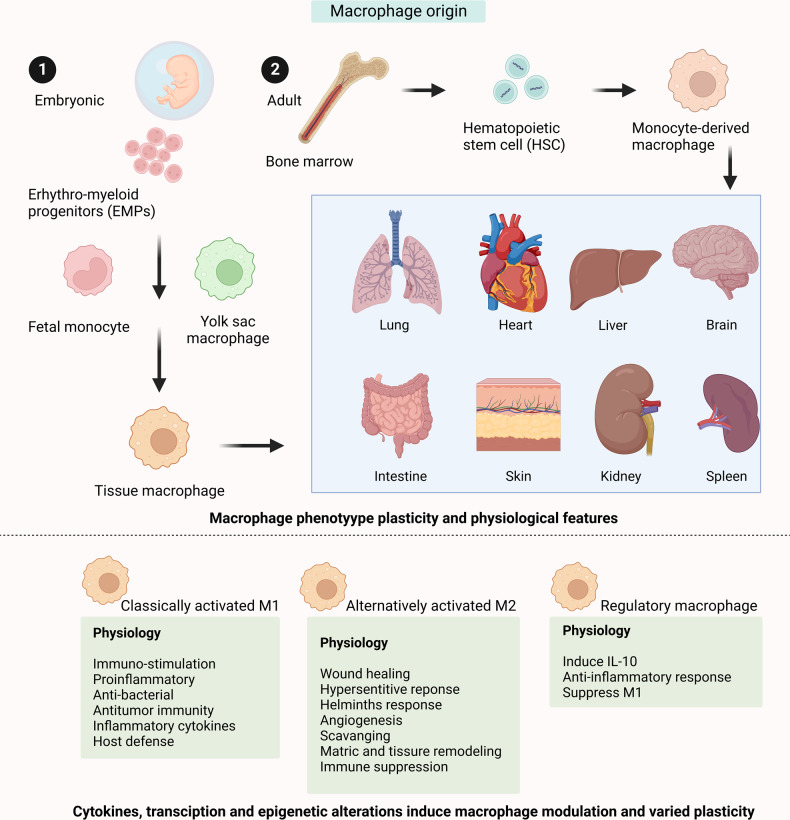
The M1/M2 macrophage origin, activation, and functional basis. Macrophages are typically produced from embryonic progenitors and involve inputs from yolk sac macrophages, blood monocytes independent, and adult monocytes originating from bone marrow. Macrophage immune modulation, functional plasticity, and phenotype changes are centered on cytokines, transcription, and epigenetic deviations

The plasticity of the macrophages assists them to adaptin the microenvironment by changing the activation state ensuing in comprehensive classification and activation of M1 or M2 macrophages (Fig. 2).^54^ Consequently, through TLR and CLR recognition, the immune cells respond to invading microbes and regenerate inflammatory cytokines, including IFN-γ, resulting in macrophage polarization to an M1 phenotype.^55^ M2-polarized macrophages can re-polarization in response to trigger M1 stimulation like IFN-γ.^56^ suggesting the imperative significance of the native cytokine milieu in leading macrophage polarization. It has been shown that IL-4 and IFN-γ can cross-regulate themselves as settings favorable to IFN-γ production for inhibiting IL-4 production.^57^ It is possible to induce the activation of M1 macrophage in a STAT1-dependent route by manufacturing IFN-γ through Th1-type T cell types and NK cells. The IL-4 and/or IL-13-induced stimulation of the STAT6 pathway triggers M2 macrophage activation.^58,59^ arginase-1 (Arg-1), out of frequent markers for M2 macrophage, reflects a context-dependent marker as it can be stimulated by STAT6 and STAT3, related to executing partial tissue reparative function of M2 macrophage.^60,61^

Important markers comprising cytokines and chemokines produced from activated macrophages can prompt the recruitment of leukocytes and infection resolution to identify macrophage activation phenotypes. Numerous M1 markers are subjected to the upregulation and expression of IFN-γ, developed by innate and adaptive immune cells, consisting of CD4^+^ Th1-type T cells, CD8^+^ T cells, and natural killer (NK) cells (46). Macrophages are skilled in manufacturing only IFN-γ and LPS or LPS in humans or IL-12 and IL-18 in mice.^62^

### Functions of activated macrophages

#### Phagocytosis

Phagocytosis is an essential function achieved by macrophages to satisfy their crucial purpose in removing disease and remodeling tissues. The impact of substrate rigidity on macrophages’ phagocytic activity has been evaluated in several investigations. RAW 264.7 macrophages and alveolar macrophages in humans present the exaggerated phagocytic function of beads using a stiffer substrate.^63^ Conversely, in Adlerz and coworker’s study, substrate stiffness didn’t disturb the phagocytic role of monocyte-derived macrophage’s fluorescent beads.^64^ In distinction, Scheraga and associates perceived stiffness with the impact on phagocytic activity in LPS-stimulated murine BMDMs and alveolar macrophages.^65^ The cell’s firmness is also obtained by inducing extracellular strain onto the cells. The work took into account the effect of these strains on macrophages. They found that PMA-differentiated THP-1 macrophages display increased combined compact phagocytic activity (+20 and +100 mmHg).

Moreover, in alternative work, it was displayed that this impact of rapidly cumulative phagocytosis by growing extracellular compression may also be discovered in primary monocytes in humans and monocyte-derived phagocytes.^66^ It is known that p38 mitogen-activated protein kinase (MAPK) is a further contributor concerned with the impact of extracellular compression on macrophage phagocytic activity.^66^

To finish with the topography of the constituent component to be consumed, phagocytosis and macrophage activation are disturbed. IL-1β production after NLRP3 activation was more significant after the absorption of textured particles than smooth surface particles.^67^ Another work delineated in the section ‘Biochemical cues’ discovered that, in contrast, surface features intrinsically might not modulate the foreign body response/reaction (FBR). The biased surface assimilation of proteins by different surfaces may account for variations in FBR.^68^ The lungs are often the central tissue location where stretches are often intimate with the cells. In response to lung infection, alveolar macrophages and lung epithelial cells release proinflammatory cytokines. IL-6, TNFs and IL-1 enhance the transcription of cell adhesion molecules (CAMs) and vascular endothelial growth factor (VEGF), improving lung endothelial penetration and decreasing the protective barrier, enabling viral dispersion and the influx of neutrophils and inflammatory monocytes.^69^ Macrophages generate NO, which may occur in the vicinity to guard alveolar cells against elasticity-induced cell damage or apoptosis in vitro. The surfactants within the lungs regulate the cellular surface tension cells within the alveoli, together with macrophages.^70^ Wu and associates sought the strategy behind stretch-induced respiratory infection comprising lung inflammation and directed in mice alveolar macrophages that ROS generation was augmented following the ventilation activity.^71^

Stretch was additionally considered in phagocyte categories that don’t seem to be directly associated with tissues of the lungs. The effect of strain on primary human monocytes following phorbol myristate acetate (PMA) treatment and U937 macrophage-like cells resulted in an augmented yield of enzymes degrading the matrix precisely, possibly instigating cellular feedback to elasticity to change the native ECM.^72^ In peritoneum macrophages, 20% static strain enhanced chemokine and cytokine production; stimulation with LPS further increased cyclooxygenase-2 (COX-2) and synergistic generation of IL-6. Applying cyclic biaxial stress and titanium elements to bone marrow-derived macrophages (BMMs) or RAW264.7 cells did not significantly influence pro-inflammatory genetic factors (but it was seen in osteoblasts).^73^ It has been demonstrated that concentrations of oxygen will affect the feedback of macrophages to elasticity: PMA-segregated THP-1 macrophages that were applied to 100% strain at 1 Hz for 24 h revealed elasticity triggered expansion and coordination; however, this result was repressed beneath hypoxic settings, at a site where expression of hypoxia-inducible factor-1α (HIF-1α) was improved.^74^

#### Regulation and evasion of macrophage autophagy

Autophagy is an effective cellular strategy of enclosing materials or pathogens in the cytoplasm into a double-membrane cellular organelle called the autophagosome, which disintegrates substrates with the help of lysosomes. The process of autophagy is coordinated into different capacities and procedures of the immune system. It is a substantial obstruction component to ensure the body’s organization against external pathogenic invaders and threat signals, assuming an essential function in the enlistment and guideline of inflammatory responses in innate immune cells.^75^ This remarkably detailed process combines more than 30 autophagy-related genes (Atgs) as operating units and immune signaling pathways. Atgs, serine/threonine kinase ULK1, and Beclin-1, in contrast to Atg14 and type III phosphatidylinositol 3-kinase Vps34 Atgs, advance the structure of a cup-shaped separation membrane to inundate the load after autophagy has been initiated. Similarly, The cell-death-inhibiting action seems to be achieved by ULK1 phosphorylation of S357 inside the intermediary motif of RIPK1. According to the study, ULK1 is a possible modulator of RIPK1-induced cell death.^76^

Macrophages perceive pathogens by surface-induced receptors, in this way, overwhelming and processing them. Macrophages with M1 increment and discharge huge measures of inflammatory factors, for example, TNF-α, IL-1, iNOS, IL-6, and several chemokines, C-C chemokine ligand 2/4 (CCL2/4) chemokine and (C-X-C theme) ligand 8/11 (CXCL8/11) chemokine, that can initiate the immunity intervened by Th1 cells, aberrant inflammation, endotoxic distress, and organ damage.^77,78^

In macrophages, xenophagy has, for the most part, been described during bacterial disease. Immunity-related GTPase family M protein in human macrophages is interested in xenophagy by advancing ROS yield and selecting autophagy apparatus after PAMP introduction. Eventually, the autophagosome sends invading intracellular microorganisms to the lysosome for disintegration.^78^ Autophagy modifies the surface expression of the phagocytic receptors apparatus and manages phagocytosis circuitously. Macrophages deficient in Atg protein Atg7 increase the upregulation of MARCO and MSR1 binary class A scrounger receptors, encouraging the phagocytosis activity of *M. bovis* BCG and Mycobacterium *tuberculosis* (MTB).^79^

Furthermore, autophagy can down-regulate inflammasome actuation through numerous components. The upregulation of IL-1β and pyroptosis is brought about by the loss of Atg7 in alveolar macrophages.^80^ Features and levels of autophagy subordinate extraordinarily to the macrophage microenvironment. The nearness of nutrient D in serum upgrades macrophage autophagy considerably by employing the induction of cathelicidin antimicrobial peptide. Moreover, human macrophages’ 1,25(OH)2D3 enhances innate immune effectors and cathelicidin production with TLR2/1 stimulation.^81^ T cells can likewise trigger an autophagocytosis response in MTB-infested macrophages in humans. At long last, microbiota may impact autophagy activity as well. Currently, the upregulation of autophagy genes in macrophages is brought about by the probiotic *Bacillus amyloliquefaciens*, which outcomes in the enhanced killing of *Escherichia coli*.^82^

A large cluster of strategies is built by intracellular bacterial pathogens to offset antibacterial resistances in macrophages, and autophagy response is no particular case. Cytosolic *L. monocytogenes* keep from engulfing via autophagy machinery by using two virulence factors, ActA and InlK. To avoid being killed by macrophages, microbial pathogens have developed complex strategies. It has proven a vital model organism to decode the molecular processes of the interactions between pathogenic bacteria and macrophages using *L. monocytogenes*, *S. aureus*, or *Yersinia spp*.^83^ Following transmission, *Francisella* evades the phagosome and enters the host cell cytoplasm, replicating extensively. Autophagy targets *Francisella* once it is in the cytosol. Autophagy’s function in this cytosolic pathogen’s replication has not been completely understood. It was observed that *Francisella tularensis* delivers a surface polysaccharide O-antigen and camouflages itself legitimately, which forestalls cytoplasmic pathogenic detection and outcomes in xenophagy. Then again, cytosolic O-antigen mutants were destroyed inside murine macrophages by Atg5-dependent autophagy.^84^ Likewise, *Salmonella* can trigger cell autophagy due to incursion, which defends cells against microbial invasion. *S. typhimurium* initiates the mammalian target of rapamycin (mTOR) and anticipates autophagy in macrophages, an ace repressor of autophagy. Prominently, macrophages deficient in FAK are progressively proficient in eliminating *S. typhimurium* disease in vivo, compared to wild-type partners.^85^

A few pathogens can repress signaling pathways that promote autophagy activation. For example, macrophages can exploit negative feedback regulatory circuits to target autophagy. For instance, NLRC4-dependent caspase-1 cleavage is prompted by *P. aeruginosa* infection that outcomes in TRIF cleavage, a significant inducer of TLR4-incited autophagy.^86^

In addition, host defense systems against pathogens, such as viruses, bacteria, and fungi, are controlled by miRNAs. MTB, the pathogen that causes TB and its host to interact, plays an essential role in determining the direction of the disease. According to a research observation, cell death, inflammatory processes, autophagy, and macrophage polarity are all regulated by the differential expression of miRNAs in the host during infection with MTB. Virulent MTB may use host miRNAs to increase pathogenicity by inhibiting host-mediated antimicrobial signaling pathways. To keep pathogens at bay, host-induced miRNAs boost antibacterial mechanisms like autophagy. The miR-125a is initiated by MTB, which advances intracellular pathogen survival inside macrophages and restrains autophagy by targeting the UVRAG complex with Beclin-1.^87^ A critical MTB virulence factor blocks autophagosome assembly in macrophages, Esat-6.^87^

## Immunometabolic regulation of macrophage activation

In the last 20 years, concentrations have given us a unique understanding of the realistic and phenotypic better macrophage variety that parallels their critical role in the host defense, homeostasis, and pathogenesis process. In addition, metabolic investigations have late revealed the significant activity associated with metabolism and metabolites in forming phenotypic changes and macrophage capacity.^88^

Usually, regulating catabolic and anabolic metabolism pathways provide energy and biosynthetic antecedents necessary for growth and structured cell-level help. A systematic analysis of immunometabolic pathways focuses on macrophages, which are central in both pro- and anti-inflammatory immune reactions and are the products of the immediate after-effect of metabolism reprogramming. Gradually, as we become familiar with the precise capacity of metabolic routes and intermediates involved in the immune function, an innovative chance to aim for immunometabolic treatment has been established.^89^

### Metabolic signaling and immune regulatory pathways

**Fig. 3:**
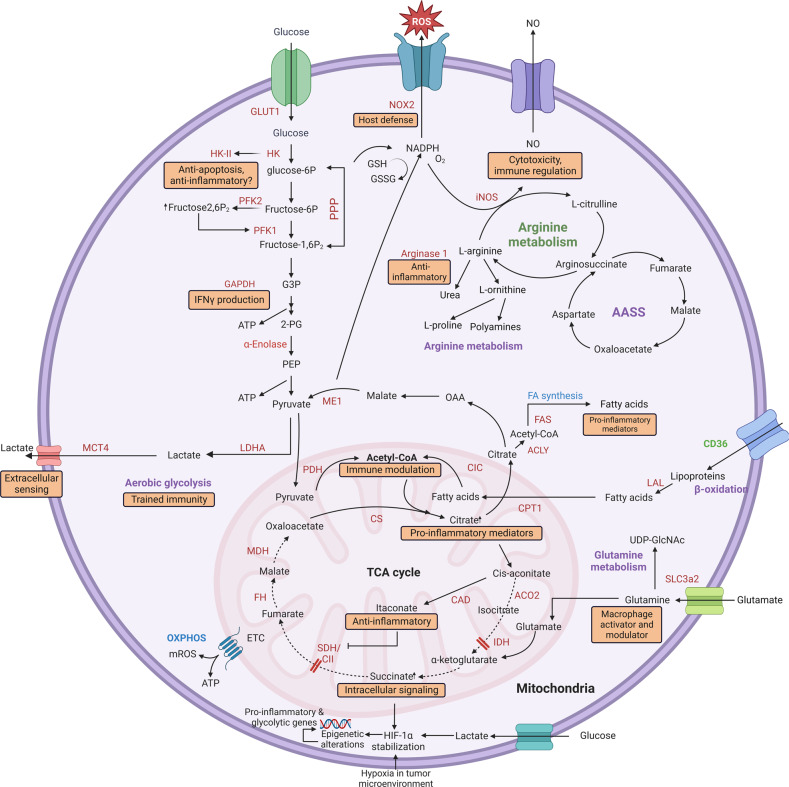
M1/M2 macrophage metabolic signaling pathways and immune regulation. M1 macrophage is featured by aerobic glycolysis, which leads to lactate development. The ROS and NO are produced accordingly. The PPP produces NADPH correlated with arginine synthesis and the aspartate-arginosuccinate shunt pathway (AASS). In addition, the tricarboxylic acid cycle (TCA) produces essential citrate and succinate vital to the metabolism of fatty acids and the stabilization of HIF-1α, leading to the transcription of pro-inflammatory and glycolytic genes and epigenetic alterations. On the other hand, M2 macrophage primarily generates ATP in an oxidative TCA cycle, combined with OXPHOS. This also metabolizes arginine. Similarly, the process depends on the energy sources of β-oxidation and glutamine metabolism. Also, precise signaling and immune regulation are vital in metabolic pathways, including aerobic glycolysis leading to lactate, NO, fatty acid synthesis, and glutamine pathways. Equally, acetyl-CoA, citrate, itaconate, and succinate are involved in immune regulation in the TCA cycle. Similarly, hexokinase 2 (HK-II), glyceraldehyde 3-phosphate dehydrogenase (GAPDH), and arginase 1 play their roles in immune regulation. All enzymes are shown in orange. GLUT1 glucose transporter 1, NOX2 NADPH oxidase 2, iNOS inducible nitric oxide synthase, HK hexokinase, PFK1 phosphofructo-1-kinase, PFK2 phosphofructokinase-2, LDHA lactate dehydrogenase A, MCT4 monocarboxylate transporter 4, ME1 malic enzyme, ACLY ATP-citrate lyase, FA fatty acids, FAS: fatty acid synthase, CIC citrate carrier, PDH pyruvate dehydrogenase, MDH malate dehydrogenase, FH fumarate hydratase, SDH succinate dehydrogenase, CII complex II, CAD cis-aconitate decarboxylase, ACO2 aconitase 2, IDH isocitrate dehydrogenase, SLC3a2 solute carrier family 3 member 2, LAL lysosomal acid lipase, CPT-1 carnitine palmitoyltransferase I, CD36 cluster of differentiation 36

Macrophages are currently viewed as significant players in immune regulation critical for health. Growing evidence reveals that M1/M2 macrophage metabolic pathways are profoundly connected to immune capacities (Fig. 3). Comprehending the connection between cell metabolic features and immune sensing signaling pathways in macrophages may assemble appropriate therapeutic methodologies for inflammatory disorders.^90^

#### Metabolic regulation in M1 macrophage

IFN-γ and LPS trigger macrophages, resulting in a TCA cycle revealed through integrated transcriptional and metabolic pathways. The TCA cycle is a constraint at the rates of isocitrate dehydrogenase (IDH) and succinate dehydrogenase (SDH), giving rise to the accumulation of concinate and citrate metabolites.^91^ Citrate build-up is the consequence of the downregulation of isocitrate dehydrogenase 1 (IDH1). The enzyme is responsible for the transfer of isocitrate (a citrate isomer) to α-ketoglutarate. The upstream-citrate metabolite itaconic acid inhibiting SDH is a crucial characteristic of macrophages that IFN-γ/LPS polarize. Itaconate is an immunomodulator with potent anti-inflammatory and antimicrobial effects on pro-inflammatory macrophages.^92^ Changes in the concentration of metabolites may directly alter signaling pathway function. HIF-1α is stabilized by the accumulation of succinate in LPS-induced macrophages, which promotes pro-inflammatory cytokine IL-1β expression.^93^ Nonetheless, stabilization of HIF-1α leads to increased glycolytic flux in M1 macrophages since HIF directly induces several glycolytic genes expression, such as monocarboxylate transporter 4 (MCT4) and glucose transporter 1 (GLUT1).^94^ Meanwhile, HIF-1 regulates innate immunity by controlling glycolysis. Lastly, HIF-1 contributes to a proliferative metabolism by regulating the glycolytic cascade, including hexokinase II (HKII).^95^ Another feature of macrophage M1 is an improved pentose phosphate pathway (PPP) that produces NADPH. In addition, NADPH is required to catabolize arginine into the NO and L-citrulline as a cofactor for iNOS induced by LPS. NADPH, in effect, produces inflammatory mediators NO and ROS. It also produces antioxidant glutathione (GSH) that preserves redox homeostasis and prevents ROS damage to cells.^96^ NO is also a critical M1 macrophage metabolism regulator that stimulates iron-sulfur-containing electron transport chain (ETC) clusters to become nitrosylated and inhibits oxidative phosphorylation (OXPHOS) and mitochondrial respiration.^97^

Cytosolic citrate is required for NADPH production and redox balance preservation. The mitochondrial citrate carrier (CIC) moves citrate from the mitochondria to the cytosol, which the enzyme ATP-citrate lyase (ACLY) transfers to the acetyl-CoA and OAA. In macrophages, the combined expression of CIC and ACLY is activated by the signaling of NF-κB and/or STAT) by inflammatory factors such as LPS, TNF-α, and IFN-γ. NADPH derived from citrate can help maintain a redox balance that connects the metabolic intermediate to the M1 macrophage pro-inflammatory functionality.^98^ In addition, the activation of chromatin-modifying enzymes (histone acetylation in HK-2, PFK1, and LDHA) may also be regulated by acetyl-CoA, which links metabolism and epigenetics.^99^ Acetyl-CoA is an additional component of the biosynthesis of fatty acids. Fatty acids are precursors for prostaglandin synthesis in TNF-α activated LPS-activated macrophages or IFN-γ.^91^

#### Metabolic regulation in M2 macrophage

The metabolic phenotype of the M2 macrophage reveals significant variations with M1 macrophages, which is consistent in terms of their distinct function as an anti-inflammatory component and a homeostasis mediator of tissue. Energy absorption is one of the significant biochemical differences between macrophages M1 and M2. While M1 macrophages ideally derive their energy from glycolysis, M2 macrophages produce ATP primarily via a typical oxidative TCA cycle with OXPHOS. This is powered by β-fatty acid oxidation (FAO) and glutamine metabolism’s anaplerotic development of α-ketoglutarate.^100,101^

Lipoproteins are essential components of fatty acids extracted from the CD36 scavenger receptor and catabolized in the lysosome by lysosomal lipase acid (LAL). In addition, carnitine palmitoyltransferase (CPT)−1a is vital for transporting long-chain fatty acids across the mitochondria. Further, glutamine is implicated by the hexosamine process in the synthesis of UDP-GlcNAc.^102,103^ UDP-GlcNAc tends to glycosylate the proteins.^104^ The upregulation and mitochondrial biogenesis of the FAO in macrophages mediated by IL-4/IL-13 is regulated via the mutual activity of STAT6, PPARs, and PGC-1β. M1 and M2 macrophages display an opposing arginine metabolism. However, M1 macrophages upregulate iNOS and metabolize L-arginine to the antimicrobials in the NO and L-citrulline. Moreover, M2 macrophages catalyze the catalyzation of L-arginine to urea and the catalyzation of L-ornithine by induction of Arg-1.^105,106^

#### Metabolic regulation in GM-/M-CSF-mediated macrophage activation

Significantly, two particular cytokines that can determine the production of functional in vivo macrophages beginning from monocytes include the colony-stimulating factor (M-CSF) macrophage and other cytokines, including the granulocyte-macrophage colony-stimulating factor (GM-CSF).^107^ Previously, GM-CSF-formed GM-BMM exhibited significantly more inflammation with higher TNF-α, IL-6, and IL-1β than M-CSF-formed M-BMM.^108–110^ Conversely, M-BMM produces more IL-10 paralleled with GM-BMM. A complete proteomic study uncovered that cellular metabolism administers macrophage function in cell pathways for integrating cytokines.^110^

Regarding extracellular fermentation, the metabolic varieties involving GM-BMM and M-BMM are envisaged through fast media dye changes by developing usable GM-BMM, which is not present in the M-BMM population. Accordingly, proteomic-related studies comparing GM-BMM and M-BMM macrophages showed that certain glycolytic compounds are strengthened than M-BMM in GM-BMM. A notable analysis of gene ontology utilizing differentially expressed proteins (DEPs), and phosphoproteins found that glucose’s metabolic/lipid-engineered/amino acid pathways are now potentiated well ahead of LPS.^111^ Since studying GM-CSF’s essential roles in that in vivo macrophage development, GM-CSF handling is shown to have enhanced LPS-instigated glycolysis in M-BMM within 12 h. Furthermore, it was theoretically linked to an inflammatory phenotype as 2-deoxyglucose (2-DG) glycolysis restriction removed GM-CSF-intervened enhancement of TNF-α, IL-1β, IL-6, and IL-12p70 mix after activation of LPS.^112,113^

Macrophages respond swiftly to environmental signals to modify their phenotype within an extensive in vivo assortment. Macrophage polarization, M1, and M2 macrophages speak to scale limits and are currently characterized as individual M-CSF-derived macrophages prepared by LPS/IFN-γ or IL-13.^114^ The metabolism of the M1 macrophages is recognized by increased glycolysis, movement over the PPP, unsaturated fat mixture, and a curbed TCA cycle that prompts the accumulation of succinate and citrate. The advancement of phosphofructokinase 2 (PFK2) transitions starts with the liver isoform (L-PFK2) to the additional complex, omnipresent isoform, ubiquitous 6-phosphofructo-2-kinase/fructose-2,6-bisphosphatase (U-PFK2) after IFN-γ/LPS activation.^46^ Another component of M1 macrophages is an improved PPP, which is robust with a glycolytic transformation of greater significance. The rapid downregulation of carbohydrate kinase-like protein (CARKL) was conducted in vitro and in vivo after stimulation of LPS.^57^

In conclusion, IFN-γ/LPS promoted M1 macrophage offers to ascend to a curtailed TCA cycle prompting the accretion of succinate and associated citrate metabolites. The development of citrate is the aftereffect of IDH1 transcriptional downregulation. Jha and associates establish that immune responsive quality 1 (*irg1*) is one of the best-upregulated genes in the macrophages stimulated by IFN-γ/LPS.^115,116^ Succinate is a significant regulator of glycolytic motion in macrophage M1, thinking that HIF initiates numerous glycolytic genes.^117^ Also, LPS diminishes the formation of AMPK in macrophages. As energy is monitored by AMPK, which is necessarily constrained, this energy-conserving enzyme hinders anabolic signaling pathways, for example, gluconeogenesis, and activates catabolic signaling pathways, comprising β-oxidation of the fatty acids. Induction of proteins associated with OXPHOS is initiated by AMPK, for instance, PGC1β, and functionalities of mitochondrial enzymatic proteins, consisting of SDH. LPS-interceded glucose take-up mTOR-HIF-1α axis influences numerous objectives to improve glycolytic signaling pathways and aids the inflammatory functions of macrophages.^118^

#### Citrulline in macrophage regulation

In both the urea cycle and the citrulline-NO cycle, the enzyme known as argininosuccinate synthetase (ASS1) is responsible for converting citrulline into argininosuccinate. It is important to note that the citrulline that is generated by M1 macrophages can be processed by ASS1 and argininosuccinate lyase (Asl) to restore arginine, which then results in the generation of NO. In the beginning phases of NO generation, macrophages primarily depend on arginine found outside of the cell to synthesize NO.^46,119^ Nevertheless, the modulation and biochemical activity of citrulline biosynthesis in the immune system are not entirely understood at this time.^46^

In a recent study, after stimulation with IFN-γ and/or lipopolysaccharide (LPS), Mao et al. discovered that citrulline levels in macrophages quickly dropped, a phenomenon necessary for the effective initiation of proinflammatory signaling. On a molecular level, citrulline depletion is triggered by IFN-γ and/or LPS activation, which in turn boosts STAT1-mediated ASS1 transcription and JAK2-mediated phosphorylation of ASS1 at tyrosine 87. Elevated citrulline, in turn, immediately attaches to JAK2 and suppresses JAK2-STAT1 signaling. The host’s defense against bacterial invasion is attenuated in vivo when ASS1-mediated citrulline reduction is inhibited. Consequently, the authors identified citrulline as an inherent immune-signaling molecule that activates a metabolic barrier for proinflammatory reactions and describes a key function for ASS1 in regulating inflammatory macrophage activation and antimicrobial defense through the reduction of cellular citrulline.^120^

#### Creatinine in macrophage metabolic regulation

Several lines of evidence suggest that creatine, a byproduct of L-arginine most well-known for its function in energy metabolism, also plays a part in immunological modulation. In particular, creatine controlled immunological reactions mediated by macrophages by regulating the proportion of their classically activated to their alternatively activated forms. Moreover, creatine constitutes a metabolic-signaling-transcriptional network that regulates macrophage effector functions.^121^

The Chen and Hu teams investigated the effects of creatine catabolism on monocyte orientation. After treating peritoneal macrophages grown from wild-type mice with creatinine, the authors showed that the intracellular content of creatinine increased, correlated with a decrease in M1 growth and a shift toward the M2 phenotype.^121^ Similarly, their previous work discovered that when mouse and human macrophage lines were co-cultured with creatinine, a downregulated product of M1 macrophages, TNF-α, was produced.^122^

### Metabolic reprogramming by pathogens and cancers

Pathogens can modulate the physiology and metabolism of macrophages by activating innate immune signaling pathways, PAMPs, or virulence factors.^123^ Generally, surface or cytosolic sensors, such as the significant PRRs in macrophages, recognize exposed microbial products (LPS, peptidoglycan, short-chain fatty acids, RNA, and cytidine-phosphate guanosine (CpG) DNA), and macrophages exhibit a shift to the Warburg-like response with the generation of lactate, ROS, and NO.^124^ The Warburg shift benefits an M1-like macrophage phenotype and antimicrobial responses.^125^ Studies showed that fungal cell wall component β-glucan could trigger a shift from OXPHOS to glycolysis with a manner of “trained immunity”.^126^ During the MTB infection process, NO can modulate macrophage activation through the activation of HIF-1α, iNOS, and repression of NF-κB.^127^ Emerging evidence proved that metabolic profiling during HIV infection in human primary macrophages presented elevated levels of ribose-5-phosphate, a critical metabolic component in nucleotide biosynthesis.^128^

In cancers, the M1-M2 programs are thought to rely principally on metabolism by various signaling pathways, including the phosphatidylinositol 3-kinase (PI3K)-AKT, c-Myc, HIF, AMPK, and PPAR pathways. Dysregulation in metabolic systems is a driving force of skewing macrophages from M1 toward the M2 phenotypical state. Pro-tumor M2 macrophages preferentially utilize OXPHOS/FAO to meet their metabolic demands, while anti-tumor M1 macrophages use glycolysis as their dominant metabolic source. Metabolic reprogramming in macrophage polarization has been extensively documented in inflammation,^129^ tissue regeneration^130^, and TME.^131^ Recently, crosstalk within and across all intratumoral extracellular compartments has been reported, and high potassium (K^+^) TME has shown immune-suppressive potency on T cells and TAMs. Di Wang’s group mechanically showed that a deficiency of Kir2.1, an essential K^+^ channel protein, disturbed macrophage absorption of K+ and glutamine uptake, stimulating TAM metabolic reprogramming from oxidative phosphorylation toward glycolysis.^132^ Other studies showed that lactate from the endothelial cell (EC) or cancer cell promotes M2-like macrophage polarization by a novel metabolic reprogramming code-histone lactylation. Moreover, cross-talks between M1/M2 macrophages with other cells (such as cytotoxic T lymphocytes, regulatory T (Treg) cells, NK cells, MDSCs, cancer-associated fibroblasts (CAFs), and cancer stem cells (CSCs)) within the TME are carried out and regulated for combat between tumor cells with cells of the immune system. For example, Treg cells selectively sustained M2-like TAM metabolic fitness. Studies showed that Treg suppresses the secretion of IFNγ in CD8^+^ T cells, which would otherwise block the sterol regulatory element-binding protein 1 (SREBP1)-mediated fatty acid synthesis in TAMs.^133^ The mechanism of tumor control over M1 macrophage metabolism is surveyed. Integrin αV/β8 on tumor cells can interact with glycoprotein-A repetition predominant (GARP) on M1 macrophages, induce macrophage DNA methylation, and downregulate gene expressions for glucose metabolism and OXPHOS in M1 macrophages.^134^ Competition also occurs between macrophages with endothelial cells (ECs). Activation of the mTOR increases glucose uptake and glycolysis in hypoxic TAMs. It promotes competition between these cells with ECs, reducing EC glucose acquisition, restoring tumor oxygenation and hampering metastasis. In addition, cancer-produced β-glucosylceramide drives the reshuffling of lipid composition on the ER membrane, leading to IRE1-dependent ER stress responses. The co-engagement of the IRE1–XBP1 and IRE1-STAT3 pathways during the ER stress response promoted pro-tumorigenic polarization and pro-survival properties of TAMs.

### Mitochondrial role in signaling

Mitochondria is an effective bioenergetic organelle contributing to energy generation and cellular redox maintenance. It fills up as an immune signaling process for various innate immunological signals.^135^ The arrangement of TLR1, TLR2, and TLR4 engages mitochondria which can enhance mitochondrial ROS generation and macrophage phagosomes.^136^ A TLR signaling adaptor named tumor necrosis factor receptor-associated factor 6 (TRAF6) can translocate to mitochondria, where it connects ECSIT signaling integrator (ECSIT), which is conserved signaling intermediate evolutionary interfaces straightforwardly with complex I of the ETC and advances ROS generation. ROS moves in phagosomes encompassing intracellular bacterial microbes and helps promote the eradication of infection. Synthetic restraint of mitochondrial complex I by metformin weakens the production of IL-1β in functional macrophages because of diminished mitochondrial ROS generation.^136^ In the advancement of inflammatory diseases, macrophages play essential roles. Activation of macrophages is not only a necessary component of host defense associated with the diseases caused by microorganisms. Still, it is also related to regulating tissue physiological conditions in inflammatory diseases such as arthritis, arteriosclerosis, and obesity. It is well recognized that immune stimulations elicit inflammatory macrophages or microenvironment settings exaggerate glycolytic metabolism and diminishes related mitochondrial respiratory activity.^137^

Current research recommends reprogramming the activated macrophage metabolic pathway as the potential therapeutic approach to cure inflammatory diseases such as microbial infectious responses, including sepsis, arthritis, arteriosclerosis, obesity, TB, and viral infection like vesicular stomatitis virus (VSV).^138^

### Mechano-transduction and modulation in macrophages

Human immune cells, like macrophages, exist in most peripheral tissues. The immune cells are distinctive in that they are subjected to a greater diversification of totally diverse mechanical and environmental conditions, and it’s so not stunning that necessary immune effector responses are modified by mechanical stimuli.^139^ Controlling the surface topography could be a straightforward technique to modulate cellular level response over cell form and stretch management from a biomaterial viewpoint. Functional modulation of macrophages, composition, and polarization to varied topography has been a topic of robust analysis for many years.^140^

Inside peripheral tissues, macrophages will expertise any leading mechanical signals resembling ECM rigidity, topography, firmness, or elasticity. Nevertheless, it rests on the precise tissue in whatever phase this mechanical indication takes part: lung macrophages are significantly exposed to cyclic flexibility. However, elasticity doesn’t function in macrophage-resembling microglial brain cells. Wherever presumably the truncated ECM rigidity regulated cellular performance, ECM/substrate rigidity disturbs macrophage instigation composition and performance. It has been displayed that alveolar macrophages modify their form and structural rigidity in substrate robustness. This happens via variations in each cortical and profound complex cytoskeleton, which, astonishingly, was appealed to be free of stress fiber development.^141^ Substrate stiffness additionally regulates the scale of the force produced within the principal advantage of a macrophage, a method critical for macrophage movement and facilitated by the signal axis including Rac GTPase, Rho-associated protein kinase (ROCK), myosin-II, as well as PI3 kinase enzyme.^142^ Besides, the elasticity and phagocytic ability of the cell is more exceptional for cells grown on solid substrates, signifying that the substrate’s flexibility modulates the macrophage’s elasticity and phagocytosis function with the help of actin polymerization.^143^ A study showed that in response to mechanical stress, periosteal myeloid-lineage cells (MCs) differentiate into CD68^+^ F4/80^+^ macrophages and secrete and activate TGF-1 to promote cortical bone growth. According to the conclusions, it was the first definite proof that periosteal bone MCs regulate cortical bone growth in response to mechanical stimulation.^144^

It has been conjointly observed that inactivated murine BMMs showed a better measure of swiftness on the solid substrate. However, once stimulated by LPS, the impact was overturned. The scale of velocity displayed to be even lesser on the rigid substrate compared to not induced macrophages on malleable plastic substrates signifying that macrophage activation might affect the stiffness-mediated properties of movement.^145^ Research using polyacrylamide-PEG hydrogels with different hardness and locations for THP-1 macrophages discriminated by PMA, which exaggerated the durability of the accumulative substrate. In non-stimulated and LPS-induced cases, BMMs formed additional cytokines (between TNF-α) on more challenging substrates.^146^

Since macrophages played their roles in tissue remodeling, they collectively took part in crucial functions within the remote body response to clinical implants. It has been witnessed that the cell form of J774A.1 mouse macrophage is being suffered from the coarseness of titanium exteriors. The rough surface of the titanium also surges cytokine and NO generation and the induction of bone morphogenetic protein-2 (BMP-2), an essential protein in the formation of bone that is needed for the correct assimilation of some grafts.^147^ Noticeably, enhanced implant style and fixation tactics to modulate local points of dynamic loading and curtailing wear fragments are impending options for the up-future existence of implanted materials.^148^ The macrophage RAW264.7 also responds to the roughness of the titanium exterior or nanotube surface layer topography, from different others, by changing the expression of BMP-2.^149^ Hotchkiss and coworkers witnessed that sleek titanium prompted an added inflammatory M1-like phenotype in macrophages; however, coarse titanium elicited an immunoregulatory M2 phenotype.^150^ In addition to the titanium, several constituents with unique topographies were utilized to investigate macrophage function. Alloys such as stainless steel and cobalt-chromium induced alterations in cytokine assembly and massive body cell development in RAW264.7 macrophages, reliant on the exterior topography.^151^ Uneven polyethylene surfaces resulted in an M2-like phenotype in murine BMMs paralleled to smooth surfaces.^152^

Similarly, substrate measurement is a vital aspect of the activation of macrophages. In a 2D and a 3D background, Bartneck and associates coordinated polylactic-co-glycolic acid (PLGA) fiber gels. Although in a 2D background, macrophages showed an enhanced yield of pro-inflammatory cytokines, and in 3D nanofiber frameworks, they exhibited an augmented fabrication of pro-angiogenic factors. Positively, the aperture dimension of 3D frameworks is engaged in modulating the macrophage phenotype.^153^ Zaveri et al. revealed properties of changeable macrophage integrin-binding activity by subcutaneously embedding polyethylene terephthalate (PET) biomaterials and unsettling the movement of integrin Mac1 (fibrinogen binding leukocyte integrin), Arg-Gly-Asp (RGD) (ligand existing in fibronectin, laminin, vitronectin, and fibrinogen). They also stated the advantage of modulating macrophage activities by suppressing integrin connections mutual to any or all cells (e.g., RGD).^154^

Fluid movement is revealed to be an additional factor in tissue macrophage role. It’s been displayed that macrophages are vital for eliminating multiplexes of the coagulation factor in circulation. In the circulation, coagulation factor VIII and von Willebrand factor (VWF) procedure multiplexes, and solely underneath cut-off flow environments of macrophages, are ready to assume this complex of VIII-VWF.^155^ In atherosclerotic plaques, macrophages exist in extraordinary quantities, considering paying to pathological progression in the disease. Similarly, the native macrophage phenotype is modulated by flow movement during an investigation where the histology of murine atherosclerotic wounds exposed to different fluidities remained considered. Macrophages within the tissue through truncated shear compression had augmented markers of M1 inflammatory macrophages. However, oscillatory shear stress-subjected macrophage regions showed an additional M2-like phenotype.^156^

### Macrophage regulation by Piezo1

In response to elevated membrane tension, stretch-activated ion channels allow ions to pass through the membrane. These channels play a critical part in detecting and transducing external physical stimuli into electrochemical activity, which influences signaling and the behavior of cells. For example, the mechanically triggered, non-specific cation channel known as Piezo1 is engaged in various developmental processes and pathological diseases.^157^

Recent research has shown that the Piezo1-regulated CCL2/CCR2 pathway and the Notch signaling cascade are necessary for macrophage enrichment in the damaged kidney. Piezo1 deletion can block the development of kidney fibrosis and epithelial-mesenchymal transition.^158^ Researchers have also shown that BM-Mφs may sense and react to structural changes in the vascular niche after irradiation damage, making them a viable therapeutic target for boosting hematopoietic restoration. Total body irradiation in C57BL/6 mice was used to test the BM-Mφs’ ability to survive and become activated. Reduced numbers of BMMs compounded the damage done by irradiation, slowing the repair of the sinusoidal endothelium and the proliferation of hematopoietic stem cells (HSCs). Irradiation did not eliminate all BM-Mφs, but the surviving BM-Mφs showed an activated M2-like phenotype. Post-irradiation, BM-Mφs, specifically CD206^+^ BM-Mφs, showed an increase in the production of VEGF-A, a cytokine critical for sinusoidal regeneration. In response to the mechanical changes in their environment caused by bone marrow ablation, BM-Mφs, particularly CD206^+^ BM-Mφs, elevated the expression of the mechanosensory ion channel Piezo1. Irradiation, Piezo1 activation, and the M2-like polarization generated by the phagocytosis of apoptotic cells contributed to the overexpression of Piezo1. Activation of Piezo1 was linked to elevated levels of VEGF-A expression, as well as NFATC1, NFATC2, and HIF-1 accumulation. The authors found that blocking the calcineurin/NFAT/HIF-1α signaling pathway attenuated the Piezo1-mediated increase in VEGF-A.^159^ Another study has shown that TLR4 signaling enhances macrophage bactericidal activity through the mechanical sensor Piezo1. Genetic deficiency of Piezo1 results in abrogation of these responses, which are triggered by a bacterial infection or LPS stimulation and involve the assembly of a complex between Piezo1 and TLR4 to remodel F-actin organization and enhance phagocytosis, mitochondrial-phagosomal ROS production, and bacterial clearance.^160^ The mechanically activated cation channel Piezo1 was investigated for its function in macrophage polarization and the detection of microenvironmental stiffness. It was demonstrated that Piezo1-deficient macrophages had improved wound healing and lower inflammation. Ca^2+^ flow is reliant on Piezo1, controlled by soluble cues, and amplified on rigid surfaces, as shown by macrophages expressing the transgenic Ca^2+^ reporter, Salsa6f. Results showed that Piezo1 in macrophages is a mechanosensor of stiffness and that its activity regulates polarization responses.^157^

A further function of Piezo1 is to control the phagocytic activity of macrophages, which then regulates the erythrocyte turnover rate. E756del is a moderate GOF Piezo1 allele prevalent in one-third of people of African heritage, and it has been shown to be closely related to elevated plasma iron levels. This study reveals a genetic risk factor for elevated iron levels in African Americans and establishes a connection between macrophage mechanotransduction and iron metabolism.^161^

### Macrophage extracellular traps and immune responses

Macrophages achieve assorted performances, including tissue restoration, homeostasis support, and immunity regulation. Ongoing investigations have exhibited that macrophages produce ETs.^162^ A few features have been ascribed to neutrophil ETs (NETs): grasping various pathogens, concealment, destroying the toxic bacterial elements of confined entities, and bactericidal movement.^163^ ETs are immune feedback responsible for cell “ETosis” to discharge net-like material, with filaments made out of cell DNA, compact with histone proteins and the cell proteins. Microbes are believed to be restrained and eliminated by ETs, yet they have also been comprised in infection pathology containing sterile inflammation and autoimmune disorders. Macrophage ETs (METs) are currently delivered in light of different microbes and keep comparative highlights to NETs. METs are created by a novel strategic cell death process (METosis) that outcomes in the arrival of strands made out of DNA and compact with cell proteins. METs respond to restrain and eradicate a few microbes yet may likewise assume a function in disease pathology.^164^

The first depiction of NETs is under the help of high-resolution scanning electron microscopy (SEM) imaging. To distinguish METs with NETs, most studies use SEM or scanning laser PMA derivation. However, the following accounts have proposed ETosis might take confocal microscopy (SLCM).^165^ Recognized extracellular strands are thick, 15–17 nm in diameter, and beaded by globular domains. Utilizing the immunofluorescence method, the researchers discovered that these beaded globular domains contain the proteins from neutrophil azurophilic, secondary, and myeloperoxidase (MPO), elastase, and gelatinase tertiary granules comprising lactoferrin. These extracellular strands did not include cytoskeleton parts or additional cytoplasmic proteins. The major auxiliary part of these filaments was DNA, exhibited by dyeing with DNA intercalating stains and the destruction of these constructs when subjected to DNase. By all accounts, the span duration of cells experiencing ETosis is variable. The first portrayal of NETs exhibited fast arrival of extracellular DNA that happened in as meager as 10 min subsequent stimulus with a few hours.^166^ DNA discharges from eosinophils within 5 min of a stimulus containing part C5a or LPS. The most extreme impact, estimated as fluorescence of a cell-impermeable DNA-staining dye, happened within 30 min.^167,168^

A variety of pathogens and chemical stimuli are described to instigate METs. For example, specific virulence factors, ESX-1, or the MTB secretion system, may explicitly induce MET discharge by human monocyte-derived macrophages.^169^ *Mannheimia haemolytica* septicity of bovine monocyte-induced macrophages prompted MET discharge in a leukotoxin subordinate way as a disease with leukotoxin-insufficient *M. haemolytica* cells did not affect the outcome in MET discharge.^170^ *Candida albicans* or *Escherichia coli* infection invigorates METosis. The expulsion of MET-associated constructs from the cells happens, employing a ROS-free strategy.^171^ Likewise, the investigation is constrained concerning why a few macrophages experience METosis while others don’t. For example, *Strongyloides stercoralis* infection evokes human monocyte-derived macrophages to produce METs, whereas mouse peritoneal macrophages don’t exhibit this capability.^172^ Treating each mouse RAW 264.7 macrophage-like cells or essential mouse peritoneal macrophages with individuals from the statin group of cholesterol-bringing down drugs results in improved MET discharge from these cells.^173^

Besides the associations among macrophages and specific microbes, different elements, including the cell condition and polarization state, may change a macrophage’s capacity to experience METosis. In different investigations, the operators appeared to advance these subsets to incorporate type I interferons (IFNs), retinoic acid, DNase, and low-mass granulocytes in systemic lupus erythematosus (SLE) patients with expanded autoantigens.^174^ Likewise, cell programming or environmental signs adjust the capacity of macrophages to finish METosis. For example, changes in the cytoskeleton activated by stress reactions may prompt METosis without oxidative stress or proinflammatory intermediates.^175^

The “NETotic complex” contains a few stages, including vacuolization, cytoplasmic and nuclear growth, enzyme linkage to DNA, membrane bulge, chromatin decondensation, and histone citrullination in cell membrane break and NET discharge.^176^ ETosis is viewed as an alternate cell pathway for cell death from apoptosis as neutrophils experiencing ETosis don’t show ordinary DNA discontinuity, need phosphatidylserine confinement to the external leaflet of the cell membrane, and are deficient of characteristic caspase initiation, all signs of cells experiencing apoptosis. ETosis is additionally eminently not quite the same as cell necrosis. In ETosis, molecular and granular membranes deteriorate together, although the plasma layer remains unbroken.^177^

Visualizing MET released from primary human macrophages in vitro may be used in immunofluorescence investigations. These findings pave the way for further characterization of these structures and comparison to ETs produced by neutrophils. Following the development of the M1 proinflammatory phenotype, human monocyte-derived macrophages (HMDM) generate METs in response to various inflammatory stimuli.^178^

### Macrophage extracellular traps in disease pathophysiology

ETs have been linked to aseptic inflammation and autoimmune disorders, in addition to their classical role of immobilizing and killing bacteria, and they have also been implicated in disease pathophysiology.^174^

Although there is a wide variety of tissue macrophages, very little is known about the many forms of macrophages and how they react to infections by releasing MET in the tissue microenvironment. Also poorly understood is the mechanism through which some macrophages acquire METosis, whereas others don’t. However, human and mouse neutrophils create NETs following infection with *Strongyloides stercoralis*; only human monocyte-derived macrophages produce METs, as reported by Bonne-Année et al.^172^ Similar findings were reported by Schorn et al.^179^ In contrast, neutrophils, basophils, and eosinophils could also produce ETs in response to monosodium urate crystals in gouty arthritis. Peripheral-blood monocytes did not discharge METs, albeit phagocytosing the crystals. Statins are a class of drugs used to lower cholesterol levels. Chow et al.^180^ found that handling mouse RAW 264.7 macrophages or primary mouse peritoneal macrophages with statins increased MET withdrawal from such cells. Still, Halder et al.^181^ could not show a similar response with human peripheral blood monocytes. If the various reactions may be attributed to the experimental settings or whether particular macrophages are fundamentally more susceptible to METosis remains unclear.

Various infectious entities and chemical stimuli may trigger METs. Wong and Jacobs^169^ outlined how human peripheral-blood monocyte-derived macrophages release MET in response to live bacterial cells and critical virulence factors, including the MTB secretion system, ESX-1. The presence of other chemical inducers, such as interferon-γ, further triggered this response. *Mannheimia haemolytica* infection of bovine monocyte-derived macrophages induced MET release in a leukotoxin-dependent manner since MET was not released by infection with leukotoxin-deficient *M. haemolytica* cells, as revealed by Aulik et al.^170^ There is some inconsistency across cell types and experimental settings. However, some publications have shown that proinflammatory mediators that drive the formation of ROS elicit METs.^170,180^ The intervention of mouse macrophage J774A.1 cells or primary mouse peritoneal macrophages with PMA, hydrogen peroxide, interferon-γ, or M-CSF did not result in MET release. Still, infection with *Candida albicans* or *Escherichia coli* stimulated METosis.^171^ In light of these findings, the investigators hypothesized that a ROS-independent process was responsible for releasing MET assemblies from these cells.

There is a limited understanding of how various tissue macrophages react to multiple infections that might trigger MET release. More research is required to determine if all tissue macrophages would be similarly affected by changes in cellular function due to polarization states and environmental cues. As the roles of METs in immunity and pathology are elucidated, further investigation is essential.

## Transcriptional regulation of macrophage activation

Macrophages display the remarkable dynamic and plastic ability to change their activation state according to their surrounding microenvironments. Therefore it requires different sequence-specific transcription factors to regulate other polarization states of macrophages in corresponding contexts.^182^ Below, we summarize some of the most critical factors which are relevant to macrophage polarization, including STAT family, NF-κB, Krüppel-like factors, IFN regulatory factors, peroxisome PPAR, HIF.^183^

### Signal transducers and activators of transcription

A group of the STATs family is a well-known transcription factor that can regulate macrophage M1 or M2 polarization. IFN-γ ligand binding to its receptor induces Janus kinase 1/2-mediated tyrosine phosphorylation and subsequent dimerization and activation of STAT1, which binds as a homodimer to the promoter of M1 signature genes.^184^ Activation of STAT3 by IL-10 and IL-6 stimulation can induce M2-associated markers expression such as IL-10, TGF-β1, and Mrc1.^185,186^ Cytokines IL-4 and IL-13 have been well established to induce M2a polarization of macrophages, which are mainly dependent on the STAT6 to regulate key M2 markers gene such as Mrc1, Retnlα, Fizz1, Chi3l3, and Ym1, and play a crucial role in Th2 related inflammatory diseases.^187,188^ Recently, Kamerkar et al. described a STAT6 targeting antisense oligonucleotide (ASO) (exoASO-STAT6), which was delivered by an engineered exosome that can reprogram tumor-associated macrophages and successfully treat the tumor.^189^

### Nuclear factor κB

Notably, NF-κB is the central transcriptional factor that orchestrates the inflammatory immune responses to various stimuli.^190^ The NF-κB system included five members: RelA, RelB, p65, NF-κB1 (p105/p50), NF-κB2 (p100/p52), and c-Rel.^191^ LPS is well used as a co-stimulus of IFNγ to promote the M1 macrophage polarization. It binds to the TLR4 and activates NF-κB, quickly expressing pro-inflammatory cytokines such as Tnf, Il1b, Il6, and Il12. This process typically involves Th1-related inflammatory immune responses.^192^ The NF-κB complexes compose p65 and p50 heterodimers with the inhibitory protein IκBα in the cytosol. Upon LPS stimulation, IκBα was phosphorylated by IκB kinase (IKK) and subsequently led to the translocation of the p65/p50 complex into the nucleus, where p65 binds to the promotor of M1 markers genes.^192^ As a central modulator of immune reaction, the actual regulatory role of the NF-κB system is far more complicated. The different homo- and heterodimerization compositions are associated with differential regulation of target genes and exhibit other effects.^191^ For instance, the p50 and p52 homodimers execute as repressors due to the absence of a transcription activation domain in RelA and RelB.^191^ For example, an in vitro study from Chiara Porta et al. reported that specific deletion of p50 in macrophage blocks the Pol II recruitment to M2-relevant gene promoters. However, it increased recruitment to M1 markers gene promoters and upregulated Nos2 and Tnf.^193^ NF-κB is an essential transcriptional modulator of both M1 and M2 macrophage polarization.

### IFN regulatory factors

Interferon regulatory factors are transcriptional factors that modulate the transcription of interferons. In mammals, nine IRF family members, including IRF-1, IRF-2, IRF-3, IRF-4, IRF-5, IRF-6, IRF-7, IRF-8, and IRF-9.^194^ IRFs play essential roles in various immune reactions, including antimicrobial immunity, T cell differentiation and activation, myeloid cell development and activation, and inflammation.^195^ IRFs also contribute significantly to macrophage polarization. IRF-3 is associated with inflammatory stimuli and contributes to the M1 macrophage polarization. MyD88 and TRIF work as adaptors to mediate the downstream signaling of TLR4.^196,197^ and activates IRF-3, which leads to the secretion of IFNs, such as IFN-α and IFN-βthrough the TRIF adaptor pathway.^196,198^ Subsequently, IFNs activate the transcription factor STAT1 to induce transcription of M1 marker genes such as CXCL9 and CXCL10 via IFN receptor (IFNAR).^196,199^ IRF-5 is another described interferon regulatory factor needed for optimal expression of IL-12 and pro-inflammatory cytokines in mice [200], thereby comprehensively regulating M1 polarization. IRF-5 can directly recruit to M1 gene promoters such as Il12b, whereas it represses transcription of M2 marker Il10.^200^

IRF-4 is a negative TLR signaling regulator in innate and adaptive immunity.^201^ In addition, evidence shows that IRF-4 functions as a critical transcriptional factor to regulate M2 macrophage polarization specifically. Mechanistically, IRF-4 mediated M2 polarization involves histone demethylase JMJD3, which is responsible for removing H3K27me3, an inhibitory histone modification. Macrophages with deletion of JMJD3 cannot polarize into the M2 but with no impairment of M1 polarization.^202^

### Peroxisome proliferator-activated receptors

Peroxisome PPARs are ligand-activated transcription factors that belong to the nuclear hormone receptor superfamily, including the three members: PPARα, PPARγ, and PPARβ/δ.^203^

PPAR-α, also known as NR1C1 (nuclear receptor subfamily 1, group C, member 1), is critical in regulating cholesterol, fatty acid homeostasis, and inflammatory gene expression in macrophages.^204^ Pallavi R. Devchand reported that upon the proinflammatory leukotriene B4 stimulation, PPAR-α-deficient mice exhibit prolonged inflammation.^205^ In M1 polarized macrophages, activation of PPAR-α inhibits the expression of several proinflammatory cytokines, such as TNF-α and IL-1β, by negatively influencing the transcriptional factors AP1 and NF-κB.^206^ In addition, Penas et al. showed that activation of PPAR-α induces the expression of M2 markers (Arg-1, Mrc1, and TGF-β).^207^

PPAR-γ were activated by the naturally generated substance such as fatty acids and the prostaglandin D2 metabolite 15-deoxy-Δ12,14prostaglandin J2 (15d-PGJ2) and well described for its role in both the M1 and M2 polarization. During the M1 macrophage polarization, the expression of PPAR-γ was downregulated, and it has been demonstrated to be a negative regulator of M1 macrophage activation by research from Mercedes Ricote.^208^ PPAR-γ impedes the M1 marker’s gene expression in part by antagonizing the activities of the transcription factors AP-1, STAT, and NF-κB.^209^ For example, PPAR-γ functions as a nuclear receptor corepressor-corepressor complex which blocks NF-κB transactivation ability on the promoter of M1 markers genes.^209^ The PPAR-γ expression can also be induced by IL-4 and IL-13 and was reported to be a direct downstream target of STAT6 and positively regulate the marker gene expression of M2 polarization, thereby contributing to the Th2 immune system responses.^210^ In addition, an exciting study from Attila Szanto showed that STAT6 could facilitate the DNA binding ability of PPARγ on its target genes promoters, including lipid metabolism and M2 polarization-associated gene, therefore playing a crucial role in obesity-associated metabolic disease.^188^ Similarly, PPAR-β/δ activity is also induced by the STAT6 activation during M2 macrophage polarization and is required for the M2 marker’s gene expression.^211^ Taken together, accumulative evidence points to an essential role of the PPARs family in governing the diverse immune functions of macrophages and relevant inflammatory diseases.

### Krüppel-like factors

Krüppel-like factors (KLFs) belong to a subfamily of the zinc-finger class of DNA-binding transcription factors.^212^ Currently, 18 mammalian KLFs were identified to be expressed in various tissues and play an essential role in different cellular processes, including macrophage polarization.^212^ In a condition of M1 polarization, both KLF2 and KLF4 can negatively regulate NF-κB mediated M1 transcriptional process. The underlying mechanism is that they can inhibit the accessibility of NF-κB with its cofactors, including p300 and p300/CBP-associated factor (PCAF), on the promoters of inflammatory genes.^213^ Ganapati H Mahabeleshwar et al. revealed that macrophage-specific KLF2-knockout mice are sensitive to sepsis and a robust inflammatory response and demonstrate enhanced pathogen clearance in models of bacterial peritonitis.^213^ On the contrary, KLF4 directly interacts with critical M2 transcription factor STAT6 to induce the transcription of M2 genes. Meanwhile, KLF4, downstream of STAT6, was generated in the M2 macrophage, forming a feedback loop to promote the M2 genetic program ongoing.^213^ Knights et al. revealed that KLF3 is a suppressor of M1 macrophage-mediated inflammation via directly repressing RELA/p65 activity and downstream proinflammatory cytokine production.^214^ KLF6 expression is responsive to both M1 and M2 stimuli conditions. M1 polarization can increase KLF6, whereas M2-driving stimuli downregulate its expression.^215^ KLF6 is required for optimal p65 binding to its M1 target gene promoters, thereby positively regulating the M1 polarization program.^216^ In contrast, KLF6 interacts with PPARγ to inhibit its induction of M2 gene transcription.^216^ Lastly, Yuan et al. provide evidence to demonstrate the vital role of KLF14 in regulating the glycolysis of macrophages and sepsis. Mechanistically, KLF14 decreased glycolysis and the production of inflammatory cytokines by inhibiting HK2 transcription.^217^

### Hypoxia inducible factors

HIF is a hypoxia sensor and a hypoxic cellular response regulator. HIF-1 is a heterodimer containing an oxygen-regulated HIF-1α and HIF-2α subunits and a constitutively expressed HIF-1β subunit.^218^ HIF-1α induced by M1 polarized cells via NF-кB dependent manner mediates transcription of iNOS.^219^ Mice with conditional deletion of macrophage HIF-1α exhibit a reduction of antimicrobial activity and failure to constrain the systemic spread of infection.^219^ In contrast to HIF-1α, expression of HIF-2α was induced in M2-polarized macrophages. HIF-2α promotes the induction of Arg-1, which can counteract iNOS activity.^219^

### c-MAF transcription factor

c-Maf belongs to the AP-1 family and functions as an essential leucine zipper transcription factor.^220^ Evidence suggests that c-Maf is required for macrophage self-renewal in the monocyte/macrophage pathway. Furthermore, c-Maf encourages IL-10 generation in macrophages while suppressing IL-12 output.^221^ c-Maf supports M2-like macrophage-mediated T-cell inhibition and tumor development by regulating numerous M2-related genes and their specific binding ability within a shared noncoding region of the Csf-1r gene.^75^

In addition to supporting M2-like macrophage polarization and stimulation, c-Maf acts as a metabolic barrier by modulating the TCA cycle and UDP-GlcNAc production. Moreover, c-Maf controls the inhibitory activity of TAMs because it is strongly upregulated in these cells.^222^ Research from Liu et al.^221^ confirmed that tumor load is decreased alongside improved anticancer T cell immunity after selective elimination of c-Maf in myeloid cells. The researchers also found that in a model of subcutaneous LLC tumors, blocking c-Maf partially removes resistance to anti-PD-1 treatment. It has been shown that c-Maf is found in human M2 macrophages/monocytes, tumor-infiltrating macrophages/monocytes, and systemic monocytes of lung cancer. These results define a model for c-Maf-mediated gene control of inhibitory macrophage polarization and raise the possibility that c-Maf may be a valuable therapeutic target in anticancer therapy.

## Epigenetic regulation of macrophage activation

Expressing a specific gene in cells relies on the corresponding chromatin epigenetic status, including DNA methylation, histone modifications, and microRNA-mediated regulation. Epigenetic regulation is essential in defining macrophage polarization without altering its sequence.^223^ First, DNA methylation for macrophage activation is investigated. DNA methylation happens at CpG DNA for gene silencing by changing the binding ability of methylation-sensitive transcription factors (TFs).^224^ DNA Methyl marks are catalytically added by DNA methyltransferases (DNMTs) can be passively or actively removed by the ten-eleven translocation (TET) DNA dioxygenases.^225^ DNMT1 is a maintenance methyltransferase and participates in the regulation of histone modifications. Studies showed that DNMT1-mediated cytokine signals negative regulator SOCS1 hypermethylation, leading to a lack of SOCS1 and high TNF-α and IL-6 expression.^226^ DNMT1 also manipulates the hypermethylation Notch1, PU.1, and KIF4 partnering with the dimethylation (H3K9me2) and trimethylation (H3K9me3) of H3K9 for M1 macrophage polarization.^227^ In addition, studies showed that DNMT3B could target the promoter of PPARγ, a transcription factor significantly involved in macrophage polarization with enrichment of CpG sites, inhibiting the expression of PPARG for a proinflammatory cellular state in obesity.^228^ In the mouse hepatic fibrosis (HF) model, hypermethylation of PSTPIP2 occurs and is mediated by DNMT3a and DNMT3b, causing a mixed induction of hepatic M1 and M2 macrophage M2 biomarkers.^229^ Similarly, TET deficiency by modulating DNA methylation and hydroxymethylation for activating macrophages with high IL-6 and IL-1B expression is also documented.^230^ Leishmania donovani infection induces methylation changes at the 443 CpG site in host macrophages, suppressing innate immunity and thereby enabling pathogen replication and survival.^231^ Recently, Lei Zheng’s group reported that DNA methylation participated in tumor-associated macrophage polarization from an M1-like phenotype to an M2-like phenotype in pancreatic ductal adenocarcinoma (PDA), blocking either GARP or integrin, suppressing tumor-induced DNA methylation of Nqo-1 gene.^134^ Therefore, DNA methylation has a critical essential role in macrophage development and activation, and it has been exploited as a potential therapeutic target for various human diseases.^232^

Gene induction or repression can also be controlled by histone methylation, depending on the position of methylation and the number of methyl groups. The methylation status of histone lysines is determined by the histone methyltransferase (HMT) activity and the opposing histone demethylase (HDM) activity. In general, gene expression is controlled by gene regulatory elements such as enhancers, promoters, and silencers. A lot of histone modifications are inclined to enrich these sequences. For example, H3K4me3 is enriched in promoters and H3K4me1 in enhancers. Gene activation is generally associated with H3K4, H3K36, and H3K79 methyl marks, while H3K9 and H3K27 regulate gene silencing and H4K20 patterns.^233^ In macrophages, inflammatory cytokine gene transcription is restrained without TLR signaling. The inflammatory gene loci are in a “poised” state with the presence of the negative histone marks (such as H3K9me3, H3K27me3, and H4K20me3) or occupied by repressors (such as nuclear receptors that recruit corepressor complexes).^234,235^ When macrophages are initially inflammatory activated, the locus that encodes inflammatory factors will be under a relatively “open” chromatin environment, the corepressors are removed from gene loci, and the concomitant reduction of negative histone marks trimethylations by using demethylases. Positive histone marks increase in the promoter region, such as H3K4me3 and H3K27Ac. Short transcription factors (TF), such as PU.1 and C/EBP family members, bind to and open the enhancers of these genes and thus “prime” them for M1 activation.^236^ Enhancers in this state are marked by PU.1, H3K4me1, and open chromatin. For example, macrophages primed by LPS have a high IL-12 p40 production which is triggered by a TLR-4-dependent event and histone H3 and H4 acetylation.^237^

Changing epigenetic regulation in macrophages would allow for the selective targeting of M2 macrophages, removing tumor-supporting TAMs while leaving tumor-inhibiting M1 TAMs alone. Many epigenetic enzyme pharmacologic modulators are presently in clinical use and may be repurposed to treat malignancies with a significant TAM infiltration. However, while a considerable study has been done on epigenetic enzymes and their modulators in M1 macrophages, substantially less is understood about the epigenetic modifications of M2 macrophages.^238^ Similarly, epigenetic regulators are another widespread component in tumor growth. Epigenetic regulators restructure chromatin assemblies, help genome packing, and alter gene expression frameworks without modifying the genome.^239^ Epigenetic regulators, including NAD-dependent protein deacetylase sirtuin-2 (SIRT), protein arginine methyltransferase 1 (PRMT1), Jumonji domain-containing protein 3 (JMJD3), MYND domain-containing protein 3 (SMYD3) and bromodomain and extra terminal (BET) proteins direct polarization of M2 by upregulating M2 markers, whereas DNMT3b, Jumonji domain-containing protein 1A (JMJD1A), histone deacetylase (HDAC)9, and HDAC3 organize the contrary impact.

On the other hand, HDAC3 is essential for producing genes that promote inflammation. When macrophages were activated with LPS but lacked HDAC3, roughly 50% of the gene expression programs that modulate inflammatory responses did not become active.^240,241^ Because of the inhibitory action of HDAC3 on the alternative activation of macrophages triggered by cytokines in vivo and in vitro, the capacity of various organs to react to inflammatory stimulation is drastically altered.^241^ HDAC3 initiates deacetylation in the tail of family-specific transcription factor-binding histones by binding to a subset of sites on the macrophage genome.^241^ Some family-specific transcription factor-binding histones work with the transcription factor PU.1 to modulate site-specific and signal-specific macrophage gene expression. Researchers Mullican et al.^241^ found that macrophages lacking HDAC3 experience upregulating alternative activation markers such as Arg-1 and Clec7a. These markers are engaged in alternative activation. This lends credence to the hypothesis that macrophages are more prone to alternate activation differentiation when HDAC3 is absent. In addition, HDAC3 is a critical regulatory element in regulating the fibrotic phenotype of macrophages. When HDAC3 is deleted in macrophages, the cells take on a different phenotype that has the potential to raise collagen levels and improve plaque stability.^240^

New research suggests lactate is more than just a “waste product” of glycolysis, as it regulates intrinsic and adaptive immune cell activity and causes drastic shifts in gene expression. A metabolic shift against aerobic glycolysis and lactate synthesis occurs in pro-inflammatory M1 macrophages, while a rise in oxidative phosphorylation and fatty acid oxidation is triggered in anti-inflammatory M2 macrophages.^242–244^ Histone lactylation (Kla) marks and their kinetics have recently been discovered by Zhang et al., and this finding raises the possibility that they play a role in controlling gene expression in M1 macrophage polarization.^245^ In the study, Zhang et al. showed that histone lactylation effectively promoted gene transcription from chromatin and revealed that lactate-derived histone lysine lactylation is a novel epigenetic remodeling. Researchers found 28 regions for lactylation on central histones in human and murine cells. Lactate, generated via glycolysis in response to hypoxia and bacterial stresses, acts as a forerunner for activating protein lactylation. The investigators used M1 macrophages subjected to bacteria as a model system to show that histone lactylation differs in its time kinetics from acetylation. Increased histone lactylation, which occurs during the late stages of M1 macrophage polarization, activates regulatory genes like arginase 1 that play a role in wound repair. The findings indicate that M1 macrophages exposed to bacterial stress harbor an internal “lactate clock” that activates gene expression to support balance. Lactylation of histones provides novel insight into the roles of lactate in a wide range of pathological states, from infection to cancer.^245^

The miRNAs consist of small molecular non-coding RNA (ncRNA) (with a span of ~22 nucleotides) capable of controlling gene expression after the post-transcription stage. Since they were discovered in 1993, the understanding of miRNA induction and its function in health and disease has developed considerably. However, the extensive comprehension of its display in inflammation and immunity endures delivering novel and thrilling possibilities for therapeutic exploration and clinical approaches.^246^ The initial studies exploring miRNA work in macrophage activation were centered around the TLR family, frequently TLR4 and its ligand LPS. These investigations uncovered an essential function for miRNA in the macrophage inflammatory reaction and presented many current research approaches and advancements in miRNA investigation. For instance, miRNAs can be organized by high throughput sequencing of Argonaute (Ago) protein immunoprecipitated RNA.^247^ In addition, there are three discrete cutting-edge sequencing strategies: high-throughput sequencing of RNA isolated by crosslinking immunoprecipitation (HITS-CLIP), also known as CLIP-Seq and photoactivatable ribonucleoside-enhanced crosslinking and immunoprecipitation (PAR-CLIP), which help cross-connected immunoprecipitation (CLIP) and assist in creating incredible steps toward global distinct miRNA targets having greater certainty. Studies can also approve these luciferase reporter findings regularly embraced with the end goal of anticipated miRNA-mRNA target interfaces.^248,249^

Recent advancements in the post-genomic period, especially in cutting-edge sequencing, have prompted the gratitude that most components in the genome produce ncRNAs. Nevertheless, out of 70% human genome, which has been translated into RNA, only 2% genome distinctively codes for proteins. However, DNA which has been neglected recently as scrap, the observation and depiction of the ncRNA transcriptome have uncovered protein-producing rRNAs and tRNAs subclasses of intensely dynamic RNAs, small nucleolar RNAs (snoRNAs), and small nuclear RNAs (snRNAs), which are essential for molecular linkage and mRNA joining mRNA translation inhibiting miRNAs, and 200 nucleotides long ncRNAs (lncRNAs) assorted mix.^250^

An expansive type of RNA fragment not involved in protein coding called ncRNAs is fit for reconstructing numerous cell capacities and, along these lines, could be utilized as target operators. MicroRNAs are small ncRNAs that function in the regulatory events of vascular degrees, and the advancement of atherosclerosis via post-transcriptional regulation of the expression of genes is broadly investigated.^251^ An extensive class of transcripts, lncRNAs, is comprehensively depicted as more prominent than 200 nucleotides long. Although many lncRNAs are precisely species-definite, their absence of conservation for a long time does not saturate a lack of functionality. lncRNAs play a significant role as regulators in the activation of macrophages (Table 2).^252^

**Table 2:** A list of human lncRNAs induced in macrophages, localization, stimuli, and brief functions

lncRNA	Localization	Stimuli	Functions	Ref.
PACER	Nucleus	LPS	Relates with suppressive p50- subunits to ease the production of COX-2.	^423^
THRIL	Nucleus	Pam3Csk4	Regulates TNF-α activation and production by interacting with the *TNFA* promoter over a composite with hnRNPL.	^424^
MacORIS	Cytoplasm	LPS + IFN-γ	Suppresses IFN-γ activity by JAK2/STAT1 phosphorylation.	^425^
MALAT1	Nucleus	LPS Lipid uptake	Interacts p65-p50 complex to avert NF-κB interaction to the promoter domains of NF-κB-triggered genes. Controls the transcription of CD36.	^426^
Lnc-MC	Cytoplasm	Macrophage differentiation	Binds with miR-199a-5p to suppress *ACVR1B* activity.	^427^
CircANRIL	Nucleus		Stimulates apoptosis following binding to PES1, damaging preRNA expression	^428^
LincRNA-DYNLRB2–2		Cholesterol loading	Leads atheroprotection over the regulation of *GPR119* and *ABCA1*.	^429^
RP5–833A20.1	Nucleus	Cholesterol loading	Regulates cholesterol transportation with the help of *NFIA* and miR-382–5p.	^427^
NEAT1	Nucleus	Cholesterol loading, Virus	Arbitrates the development of paraspeckles-resulting in multiple downstream responses.	^430^
IL1β-eRNA, IL1β-RBT46	Nucleus	LPS	Controls the expression of IL-1β and CXCL8 as eRNA.	^431^
Linc00689	Cytoplasm	TNFα	Codes for TNF-α-responsive micro peptide inside the HeLa cells.	^432^
LncRNA-ACOD1	Cytoplasm	Viruses	Interacts right to GOT2, augmenting its function.	^433^
FIRRE	Nucleus	LPS	Alleviates inflammatory genes with the help of hnRNPU.	^433^
CARL/Carlr	Nucleus Cytoplasm	NF-κB	Enables the transportation of p65 leading from the cytoplasm to the nucleus.	^434^
H19	Nucleus	oxLDL	Collaborates with miR-130b through foam cell establishment.	^435^
HcircRasGEF1B	Cytoplasm	LPS	Positively controls the permanence of ICAM-1 transcript and induction in the LPS/TLR4 signaling pathway	^436^
Lnc-BM	Cytoplasm	OSM	Stimulates BCBMs by arbitrating communication among breast cancer cells and the brain microenvironment	^437^
LncRNA-KCNQ1OT1	Nucleus	PMMA	Prompts M2 macrophages polarization to enhance particle-induced osteolysis by preventing miR-21a-5p	^438^
lncRNA-ATB	Cytoplasm	TGF-β1	LncRNA-ATB interacts with miR-200c to release ZEB1, instigating enhanced EMT development	^439^
FIRRE	Nucleus Cytoplasm	LPS	Relates with heterogeneous nuclear ribonucleoproteins U, controlling the permanence of mRNAs of particular inflammatory genes	^440^

Recent research has demonstrated that microRNAs control macrophage expansion by interacting with macrophage progenitors such as HSCs, which regulate the macrophage response to malignancy. Through the downregulation of proapoptotic proteins such as BCL2 killing factor 1 (BAK1), BCL2 modifying factor (BMF), and Krueppel-like factor 13 (KLF-13), miR-125a may control HSC survival and implantation.^253^ Through modulation of the PI3K/AKT pathway, miRNA-126 expression in HSCs suppresses cell cycle progression and hematopoietic output.^254^ Recently, Yin et al. observed that CD32 was the target of miR-224–5p deficiency, which activated the p65/NF-кB pathway and favored M1 macrophage polarization in osteoarthritis (OA) progression.^255^ miRNAs may indirectly regulate macrophage formation through HSCs and influence immune-related responses.

## Regulation of macrophage activation in human diseases

### Macrophages in autoimmune diseases

Macrophages play a vital role in the pathogenesis of many autoimmune disorders due to their wide range of immuno-modulatory, inflammatory, and tissue-repairing activities. These cells secreted a variety of cytokines and chemokines, which activate and attract more immune cells to the site of diseases. However, the adaptive immune system is crucial for pathogenesis in many autoimmune disorders because of autoantibodies and autoreactive B and T cells, yet, it may not be sufficient to explain why autoimmune diseases arise and the innate immune system may have an essential and unique role in the onset of autoimmune diseases. Macrophage infiltration is often seen in various autoimmune disorders.^256^

In many autoimmune disorders, it is still unclear what role macrophages play, whether they trigger disease or promote disease development, and whether their phenotype and function changes are pathogenic or just epiphenomenal. Furthermore, their diverse populations across autoimmune disorders are hardly unexplored. By better understanding, the role of macrophages in autoimmune conditions and the processes involved, novel treatment approaches may be developed in the future.

#### Systemic lupus erythematosus (SLE)

Modulating the adaptive immune system is a one-way macrophage contributing to SLE’s pathogenesis. B cell activation, plasma cell differentiation, antibody production, and isotype-switching are all humoral immune responses that cannot be activated without the co-stimulatory molecule CD40 binding to its ligand CD40L.^257^ More CD40L-expressing peripheral macrophages were seen in SLE patients than healthy controls.^258^ Consistent with this discovery, recombinant CD40L greatly enhanced the synthesis of total IgG by SLE B cells but not normal B cells.^259^ However, SLE patients and normal control B cells exhibited equivalent CD40 expression levels. Furthermore, mouse studies have shown that CD40L overexpression might produce lupus-like autoimmune illness and that CD40L neutralization inhibited autoreactive B cell activation and autoantibody generation in lupus-prone animals.^256,260,261^

Consequently, macrophages may contribute to SLE patients observed B cell hyperactivity through the CD40/CD40L signaling pathways. In addition, macrophages from SLE patients tend to develop into dendritic cells with elevated CD86 expression after being stimulated by IFN-α in the serum. These dendritic cells can deliver autoantigens to autoreactive T cells and B cells.^256,262^

#### Rheumatoid arthritis (RA)

One of the most prominent features of RA is macrophage penetration into the synovia. In RA patients, there is abundant evidence that both the frequency and the total number of macrophages are significantly elevated in synovial tissues.^256^

Unknown immune-regulatory mechanisms underlie drug-free remission in RA. Synovial tissue macrophages (STM) were recently hypothesized by Alivernini et al.^263^ to aid in maintaining joint homeostasis during remission. Phenotypic alterations in early/active RA, treatment-refractory/active RA, and RA in prolonged remission were detected by profiling 32,000 STMs using single-cell transcriptomics. Nine phenotypic clusters among four distinct STM subpopulations with varying homeostatic, regulatory, and inflammatory roles were associated with variable frequencies in each clinical condition. Two different STM subpopulations (MerTK^+^ TREM2^high^ and MerTK^+^ LYVE1^+^) with distinct remission transcriptomic signatures enriched in negative regulators of inflammation were identified by combining this cellular atlas with deep-phenotypic, spatial, and functional analyses of synovial biopsy fluorescent activated cell sorted STMs. These STMs stimulated the repair response of synovial fibroblasts in vitro and were influential makers of lipid mediators that suppress inflammation. There was a higher risk of disease flare when therapy was stopped in patients with a low percentage of MerTKpos STMs in remission. Hence, therapeutic manipulation of MerTKpos STM subpopulations could be an option for treating RA.

In another study, Zhou et al. found that ten immune cells drastically differ in RA and healthy control (HC). Combining the results of two sets of DEG screening, they acquired 202 differentially expressed genes (DEGs). The study found a positive association between CCL5 and M1 macrophages, consistent with previous research on the relationship between immune cells and biomarkers. Therefore, CCL5 may be a biomarker for diagnosing RA.^264^

#### Systemic sclerosis (SSc)

Systemic sclerosis (SSc) is a chronic autoimmune disease with unknown etiology characterized by vascular injury, activation of innate and adaptive immunity, and tissue fibrosis in multiple organs.^265^ Likewise other autoimmune disorders, women have a relatively higher risk than men of systemic sclerosis, and it has a peak of suffering in the fifth decade of individual life.^266^ So far, the exact factors to cause systemic sclerosis are mainly unknown. However, environmental factors, together with individual genetical background, must be to be involved in its etiology.^266^ Accumulative evidence indicates that innate immunity, especially macrophage activation, plays an essential role in the pathogenesis of SSc.^267^ For instance, Ishikawa et al. found macrophage infiltration to skin adnexa and collagen bundles using SSc patient’s skin specimens.^268^ Monique Hinchcliff and Diana M Toledo et al. identified a high level of CD163-positive macrophage in patients with SSc. They demonstrated that macrophage and monocyte signatures are correlated with the inflammatory gene expression signature in the skin of patients with SS.^269^

It has been suggested that M2 macrophage polarization is predominant in SSc.^267^ Of note, M2 polarized macrophage can promote the activation of fibroblasts, thereby promoting the progression of fibrosis by releasing profibrotic factors, including the TGF-β, platelet-derived growth factor (PDGF), and CCL18.^267,270,271^ Recently, Xia Gao et al. using single-cell RNA sequencing, demonstrated that Secreted Phosphoprotein 1(SPP1) expressed with CCL18 is enriched in macrophages from SSc lung tissue.^272^ It has been shown that SPP1 is a pro-fibrotic factor that can promote lung fibroblast transdifferentiation, migration, and activation.^273^ Although initial studies suggested macrophage from SSc patients mainly polarized into M2 phenotype, recent data indicate macrophage sharing both of M1 and M2 signature, for instance, a double positive of the M2 markers (CD204, CD163, CD206) with the M1 markers (CD80, CD86, TLR4) was observed by flow cytometry from SSc patients.^274–276^ Collectively, macrophages from SSc display a more complex activation profile. Therefore, it is necessary to investigate further the polarization state of macrophages in different SSc stages and clarify its exact role in SSc.

### Immune regulations of macrophages in cancers

Cancer immunotherapies utilizing immune cells in anti-cancer therapy are practical tools in the battle against cancer and are gradually being used in clinics. Nevertheless, little or no progress has been seen for most patients with solid tumors, presumably owing to the unavailability of sufficient strategies capable of reprogramming the local immunosuppressive tumor milieu and boosting antitumor immunity. Furthermore, TAMs, which increasingly invade most solid tumors, may lead to tumor progression by inducing proliferation, angiogenesis, metastasis, and forming a barrier toward antitumor immunity.^277^

#### TAM introduction and characterization

**Fig. 4:**
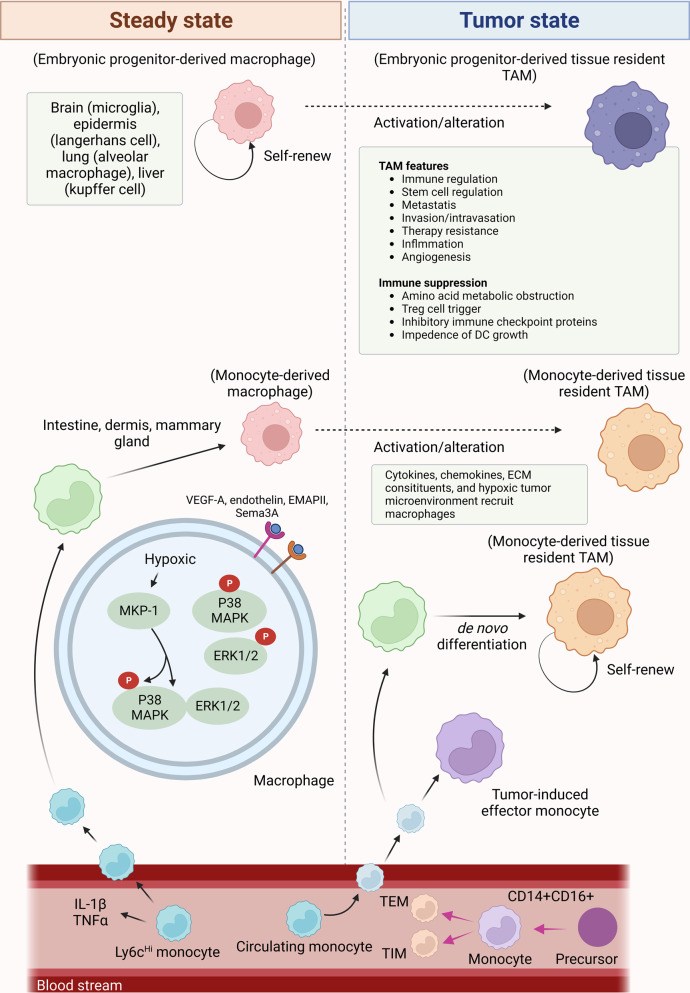
Genesis, diversity, and features of Tumor-Associated Macrophages during tumor progression and growth. Tissue-resident macrophages are derived from embryonic progenitors or HSC-derived circulating monocytes for the steady-state duration. In addition, numerous monocyte subpopulations assist in intruding myelogenous cells such as TIM, TEM, and TAM into the tumor (200). During tumor progression, TAMs may instigate through embryonic/monocytic tissue-resident macrophages activated or phenotypically altered in the course of carcinogenesis (tissue-resident TAMs) or response to tumor growth (tumor-induced TAMs). Monocytes can also directly infiltrate tumor tissue as tumor-induced effector monocyte. TAMs recruit macrophages by inducing various transcriptome and cell surface markers from a subpopulation of macrophages and embracing different pro-tumoral functions based on the TME. Such activities cause tumor initiation by inflammation, tumor progression to malignancy by stimulating angiogenesis, immunosuppression, invasion, intravasation, tumor cell extravasation at remote sites, and obstinate development of tumors. TAM tumor-associated macrophage, TEM Tie-2 [angiopoietin-2 (Ang-2)] expressing monocyte, TIM tumor-infiltrating monocyte, VEGF-A vascular endothelial growth factor A, EMAPII endothelial-monocyte-activating polypeptide II, Sema3A Semaphorin-3A. T_reg_ regulatory T cell, DC dendritic cell

Macrophages in immunity are a significant type of adaptable immunocytes, performing an expansive range of capacities ranging from modulating tissue homeostasis, defending contrary to pathogens, and assisting injury recuperation. Macrophages penetrate tumor-affected tissues or infiltrate the microenvironment of various types of solid tumors characterized as TAMs. The cell inception of macrophages, diversity, and features of TAMs and tumor progression are displayed in Fig. 4. As an essential segment of the TME, tumor advancement, metastasis, tumor angiogenesis, regulation of the immune system, and chemoresistance is influenced by TAMs. A large portion of the TAMs assembles in the central boundary and avascular domains, although there are a few additional adjustments along the vessels and abluminal side.^62^ It is accepted that bone marrow-derived HSCs fabricated circulatory blood monocytes are the essential asset of macrophages. Nevertheless, late proof recommends that a more significant part of inhabitant macrophages come from the precursors of the yolk sac, which multiply or separate in situ and produce diverse offspring for an incredible duration, for example, Kupffer cells (KCs), brain, and alveolar macrophages. These cells are selected and enacted by different cellular signals in the TME and display dramatic effects on tumor movement and metastasis.^278^

TAMs assume multi-practical functions in tumor advancement, including disease commencement and progression, immune regulation, metastasis, and angiogenesis. For instance, TAM-inferred fiery cytokines IL-17 and IL-23 have appeared to activate tumor-evoked inflammation, which leads to the imitation of tumor development. Another investigation showed that the expanded TAM-inferred IL-6 has an intensifying impact on the inflammation responses, advancing the event and improvement of hepatocellular carcinoma as liver cancer employing STAT3 signaling. Besides, TAMs adapt an M2-like macrophage phenotype and support tangible support on tumor advancement and metastasis for their delicate antigen-introducing capacity.^8^

TAMs display either polarization phenotype the same as M1/M2. Analysts, in general, consider TAMs as M2-like phenotype-procured macrophages. To a great extent, the accretion of macrophages in the TME is connected with more dreadful disease results. Classification and recognizable proof of TAMs are mainly related to their activity, such as angiogenesis, metastasis, and immune system regulation. The expression of CD204, CD68, HLA-DR, and CD14 is utilized to identify macrophages and different proteins. For example, STAT-3, CD206, B7-H4, MMP2/9, and CD163 have been used for the order of recognition of TAMs.^279^ Different microenvironmental cytokines control the polarization of TAMs, growth components, chemokines, and mixed-signal obtained from stromal cells and tumors. Surrounded by those variables, CCL2 and CSF1 are the two well-recorded M2-stimulating factors and macrophage recruiters.^280^ Another incredible pro-tumor factor is VEGF-A. In addition to its pro-angiogenic impacts, VEGF-A enhances the malignant development of tumors by instigating TAM invasion and M2 polarization within the prospect of IL-4 and IL-10. The signaling of epidermal growth factor receptor (EGFR) not only advances the expansion and obtrusiveness of tumor cells legitimately but also exploits M2-like polarization and macrophage recruitment to regulate changes in TME.^281^ Currently, numerous novel homeostatic-associated factors have been portrayed as inducers of TAM. CSF-1 allies with prostaglandin E2 (PGE2) to advance M2 macrophage polarization. CCN3 (otherwise called NOV, nephroblastoma overexpressed) prompted improved M2 macrophage invasion, though CCN3 lack delayed xenograft survival in prostate cancer growth. Moreover, chemokines and cytokines, such as CCL7, CCL8, CCL9, CCL18, CXCL12, IL-4, IL-6, and IL-13, are likewise profoundly communicated in tumors progress and associated with polarization and recruitment of TAM.^239^

#### Initiation and progression

Tumors skew the natural inclination of macrophages to inhibit proliferation, angiogenesis, and metastasis. Macrophage suppressive capability, mediated by hypoxia and fibrosis in the local microenvironment, accounts for most of the reported effectiveness. Therefore, tumor macrophages can decrease T cell recruitment and activity and modulate other tumor immunity elements. Macrophage targeting is now being studied because of the growing importance of cancer immunotherapy.^282^

High mobility group box 1 (HMGB1), heat shock proteins (HSPs), and ATP are examples of DAMPs that are released when cells die in tumors.^283,284^ Anti-tumor immunity may be boosted, for instance, when dendritic cells and macrophages are activated due to this stimulation. However, prolonged activation causes immunosuppression via the induction of IL-10, which downregulates the production of inflammatory cytokines and promotes the development of Tregs.^285^

Some proinflammatory cytokines, such as IL-6, IL-1β, and TNF-α, are secreted by macrophages, which may contribute to tumor-promoting inflammation. At the same time as it may stimulate the immune system, it also promotes the proliferation and survival of cancer cells. When TNF-α binds to TNFR1/2, NF-κB signaling is triggered. By regulating the expression of target genes (including VEGF and IL-6) and stimulating neoangiogenesis,^286^ NF-κB also promotes cancer cell proliferation and survival. By acting on the JAK/STAT3 pathway, IL-6 causes cells to proliferate, differentiate, and eventually die off (apoptosis).^287^ The pro-inflammatory cytokine IL-1 stimulates endothelial cells to create VEGF, which promotes angiogenesis and hence aids tumor invasion and dissemination. It also stimulates the production of IL-6, TNF-α, and TGF-β, promoting tumor growth.^288^ Activated macrophages generate TGF, which has dual, pro-, and anti-inflammatory roles.^289,290^ TGF-β induces apoptosis and suppresses cell cycle progression in early tumor growth. TGF-β promotes tumor invasion and metastasis by inducing epithelial-mesenchymal transition (EMT). The anti-tumor T-cell response is dampened by elevated TGF-β levels.^291^ That’s why TAMs’ inflammatory activity, especially a persistent low-grade inflammatory state, may promote tumor growth and progression.

##### Immune regulation in tumorigenesis

It has been established that TAMs decrease the cytotoxicity of T cells and NK cells because they express PD-L1, which are ligands for the programmed cell death 1 (PD-1) and CTLA-4 receptors.^292,293^ TAM-derived chemokines and cytokines may interact with bone MDSCs, tumor-related DCs, and neutrophils to create an inhibitory TME.^294^ TAM-produced IL-10 and TNF-α further decrease antitumor T cell function by inducing the expression of PD-L1. By releasing Arg-1, iNOS, oxygen radicals, or nitrogen species, TAMs may also suppress CD8^+^ T cell proliferation.^295^ To bring in Treg cells, TAMs release anti-inflammatory chemokines such as CCL2, CCL3, CCL4, CCL5, and CCL20.^296^ In addition, TAMs influence Tregs by producing CCL22 to decrease T cell-specific activity and encourage the development of cancer cells.^297^

Liu et al.^297^ conducted microenvironment characterization using multi-omics markers and found that TAM-enriched HCC tissues were linked to immunosuppression. To increase the effect of TNF-α related apoptosis-inducing ligand (TRAIL) on tumor cell death, Eisinger et al.^298^ showed that targeting an immune-suppressive TAM subtype with specific antibodies against the scavenger receptor MARCO resulted in the phenotypic conversion of TAMs into proinflammatory TAMs that recruited and activated more NK cells. Sonic Hedgehog (SHh) signaling, as emphasized by Petty et al.,^299^ increased TAM polarization by suppressing CD8^+^ T cell recruitment by preventing CXCL9 and CXCL10 production by TAMs. Finally, results support that TAMs play an immunomodulatory function that may aid tumor growth by influencing immune response and facilitating immune evasion.

##### MicroRNAs in tumorigenesis

Moreover, in tumor cell microenvironments, a lack of miR-21 causes macrophage polarization to the M1 phenotype via IFN-γ induced STAT1 signaling. Increased STAT1 signaling and PD-L1 production in macrophages, suppressing macrophage antitumor activity,^296,300^ may be achieved by downregulating miR-21. In addition, miR-127 boosted macrophage activation by downregulating M1 marker genes and upregulating M2 markers (transcription).^301^ Through the downregulation of BCL6, the expression of the phosphatase Dusp1 is suppressed when LPS induces increased miR-127. Increased inflammation and the M1 phenotype are promoted by downregulating Dusp1. In addition, miRNAs may modulate macrophage polarization toward the M2 state. Knocking down miR-124 elevated the expression of M1 indicators (i.e., CD86, TNF, and iNOS) and suppressed the expression of M2 markers (i.e., Ym1 and CD206) in M2-polarized macrophages, as shown by Veremeyko et al. Hence, the therapeutic promise of miRNAs in inflammatory disease treatment is based on their ability to regulate macrophage polarization.

Another research showed that the miR-3061/Sani1 axis might be the potential target of macrophage polarization and clarified that hyperglycemia enhanced sepsis-induced intestinal damage by boosting M1 macrophage polarization.^302^ NF-κB signaling was shown to be strongly connected with miR-22 in glioma TAMs, and the overexpression of miR-22 in macrophages was shown to suppress glioma growth in vivo. These results highlight the importance of miR-22 in macrophage phagocytosis of tumor cells and improved T cell priming, opening the door to more studies on phagocytic regulation for optimizing the response to tumor immunotherapy.^302^

It was also shown that miR-182 in macrophages causes tumor-induced M2 polarization and may be targeted for therapeutic macrophage reprogramming. The research revealed that knocking off miR-182 either constitutively in host mice or conditionally in macrophages reduced the number of M2-like TAMs and slowed the growth of breast tumors. Reconstitution of miR-182-expressing macrophages enhances tumor development, whereas targeted reduction of macrophages in mice prevents the impact of miR-182 deficiency on tumor progression. MiR-182 is directly suppressive of TLR4, which leads to NF-κB inactivation and M2 polarization of TAMs; this mechanism is triggered by cancer cells inducing miR-182 expression in macrophages through TGF-β signaling. These results highlight a critical TGF-β/miR-182/TLR4 axis for TAM polarization and support using RNA-based therapies aimed at TAM targeting in cancer treatment.^303^

As a different type of epigenetic regulator, the miRNA is likewise responsible for macrophage polarization. Until this time, miR-9, miR-125, miR-378, miR-155, miR-21, miR-187, miR-146, miR-222, miR-147, and miR-let7b are accounted for as overwhelming modulators of TAM.^304^ For instance, miR-222–3p, intent as a tumor inducer in various tumors, downregulates SOCS3 to initiate macrophages to the M2 phenotype, a JAK/STAT signaling immune pathway negative feedback regulator.^305^

##### Cancer promotion and advancement

Chronic inflammation may be linked to tumor beginning since it was shown that there were many inflammatory cells in tumor biopsy samples.^306,307^ This is true for gastric and colon cancer.^308^ This is because oncogene activation or chronic inflammation (from infection or exposure to irritants) may trigger the production of pro-inflammatory transcription factors, including NF-κB, STAT3, and HIF-1α. To attract macrophages, cancer cells may produce cytokines and chemokines (TNF-α and IL-6), which may activate these factors.^309^

The production of a mutagenic microenvironment aids cancer development by macrophages, which may release inflammatory mediators like IL-6, TNF, and IFN-γ, growth factors like epidermal growth factor (EGF) and Wnt, proteases, ROS, and nitrogen compounds.^310^ Grivennikov’s group found that TAM-derived IL-17 and IL-23 were associated with colon cancer development and progression.^311^ Kong et al.^312^ found that IL-6 produced by TAMs promoted HCC growth by activating the STAT3 signaling pathway, suggesting that IL-6 was involved in HCC formation. To sum up, TAMs may play a wide variety of roles in the onset and progression of cancer.

#### Invasion, metastasis, and angiogenesis

The spread of cancer via invasive cells and distant organs is the leading cause of mortality. Because of their enhanced motility and the degradative enzymes they produce, cancer cells can break away from the initial tumor and invade other places, where they may develop new tumors. EMT refers to the process through which epithelial cells acquire mesenchymal characteristics and acquire malignant biological traits such as invasion and metastasis.^313^

All through the EMT process, tumor cells give up cell-to-cell intersections and apical-basal polarity because of E-cadherin suppression and secure an adaptable phenotype of mesenchymal cells.^314^ Naturally, macrophages partake in the EMT procedure by discharging different dissolvable factors, for example, TGF-β, TNF-α, IL-1β, and IL-8.^315^

Recent investigations have shown that TAMs enhance metastasis and help regulate the EMT process. According to Wei’s research, TAMs boost the invasion and metastatic potential of CRC cells by inducing an EMT. Furthermore, CCL2 production upon activating this axis may aid in macrophage recruitment.^316^ High TCF4 expression was also linked to macrophage recruitment and polarization in metastatic locations. In addition, it was shown that the CCL2/CCR2 signaling pathway promoted metastasis.^317^

Co-culture experiments were conducted by Lee et al. using TAMs and non-neoplastic MCF10A human breast epithelial cells. It was shown that TAMs might release CCL2, which induced MCF10A to develop an EMT and an invasive phenotype by increasing endoplasmic reticulum oxidoreductase-1 (ERO-1) and matrix metalloproteinase-9 (MMP9).^318^ Similarly, TAM-secreted CCL5 may significantly increase prostate cancer cell invasion, metastasis, and EMT through activation of the β-catenin/STAT3 signaling pathway.^319^ With the help of CCL5 binding to CCR5 in macrophages, malignant phyllodes tumor could attract and repolarize TAMs, activating the AKT signaling pathway. Myofibroblast differentiation and invasion were further aided by TAM-generated CCL18 binding to the myofibroblast receptor PIPTNM3.^320^

It was observed by Lan et al. that CCL26, when combined with CCR3, might cause TAM invasion. CCL26 upregulation by phosphatase of regenerating liver-3 (PRL-3) promoted TAM infiltration, invasion, and metastasis in CRC.^321^ TAMs co-cultured with NSCLC cells produced conditioned media that promoted tumor cell invasion through EMT and B-Crystallin (CRYAB) overexpression, which induced lung cancer metastasis in vivo.^322^ According to Han’s results, TAMs promote osteosarcoma metastasis and invasion by increasing the production of COX-2, MMP9, and phosphorylated STAT3, which induces EMT. Some TAMs express EMT-inducing substances, such as TGF-β and IL-6. In addition, it has been shown that TAMs release EGF, which may induce EMT by activating the EGFR/ERK1/2 signal pathway in cancer cells.^323^

The M2 macrophage expresses chitinase 3-like protein 1 (CHI3L1), advancing breast cancer and gastric cells.^324^ A system upsetting macrophage activities by genetic strategies lessens the tumor cell’s endurance in pulmonary vessels and annuls tumor penetration into the lung.^325^ Selected macrophages trigger the PI3K/Akt survival signaling pathway in recently scattered breast cancer cells by drawing in vascular cell adhesion molecule-1 (VCAM-1) employing α4 integrins.^326^

It is accepted that metastasis isn’t essential to be an advanced late activity in tumor progression. Auxiliary body organs are sufficiently primed by the primary tumors and direct organ-explicit dispersal before entering tumor cells. Moreover, those “prepared” destinations are inclined to metastasis and are presented as the idea of pre-metastatic niches (PMNs). PMNs are efficiently organized and determined by essential macrophages. They were prepared for the circulation system and then bunched in the pre-metastatic destinations by an assortment of tumor-derived factors, such as exosomes, CSF-1, CCL2, TNF-α, VEGF, TGF-β, PLGF, and tissue inhibitor of metallopeptidase (TIMP).^327^ Moreover, the tissue-resident macrophages, for example, osteoclasts, pulmonary alveolar macrophages, and liver KCs, were likewise associated with organizing PMN development upon incitement. Besides, macrophages similarly build up associated metabolic cross-talk with immune cells like dendritic cells and Th1 cells and suppress their related tumoricidal and additional tumor antigen-exhibiting features, advancing the flourishing of those recently held-up tumor cells in a strategy for immunosuppression.^328^

#### Angiogenesis

TAMs may indirectly impact tumor development by increasing angiogenesis and their potential to promote inflammatory processes connected to cancer. The increased oxygen and food requirements of cancer cells need the initiation of angiogenesis.^329^ Neovascularization included a wide range of factors like hypoxia, hyperosmotic pressure, and angiogenic factors like VEGF, TGF-β, COX-2, placenta growth factor (PGF), fibroblast growth factor (FGF), angiotensin (Ang), and chemokines, is essential for tumor invasion and metastasis. Tumor cells expressed HIF in hypoxic regions, which produced pro-angiogenic molecules (including VEGF-A and FGF-2). Consistent with these observations is the discovery that HIF-1 may stimulate VEGF expression in hypoxic glioma.^330^

Yin et al.^331^ found that EGF released by TAMs could activate the EGFR on the surface of tumor cells, thereby increasing VEGF/VEGFR signaling and helping ovarian cancer cells proliferate and invade. TAMs were shown to stimulate tumor angiogenesis by Cui’s group through increased TGF-1 and IL-10 production, stimulating endothelial cell proliferation.^332^ Indirectly aiding angiogenic invasion, TAMs produce proteases such as MMP9, MMP2, and MMP3, which allow them to destroy ECM.^333^ Since an abnormal Wnt/β-catenin signaling cascade promoted cancer formation,^334^ it is clear that this route plays a role in cell proliferation, apoptosis, invasion, and metastasis. TAMs were shown to increase the expression of Wnt7b (a member of the Wnt family of ligands), which may encourage tumor neovascularization.^335^

#### Recurrence and CSC

The ability to self-renew and give rise to a diverse population of tumor cells distinguishes CSCs from other tumor cells.^336^ Wan et al.^337^ discovered that TAMs, via STAT3 signaling, may produce IL-6, promoting HCC stem cell growth. TAMs generate chemokines, including CXCL8 and CXCL12, which may instruct cancer cells to acquire a CSC-like character and sustain stemness in oral squamous, HCC, and renal cell carcinoma.^338^

The association between hyaluronic acid (HA) (the ligand of CD44) and CD44 was enhanced by HAS2 in TAMs obtained from patients with head and neck squamous cell carcinoma, as demonstrated by Gomez’s group. The PI3K-4EBP1-SOX2 signaling pathway was activated when HA coupled to CD44, which enhanced stemness.^339,340^ TAMs secrete milk-fat globule-epidermal growth factor-VIII (MFG-E8), which activates STAT3 and the Shh signaling pathway in CSCs, resulting in CSCs exhibiting treatment resistance and enhanced tumorigenicity.^341^

The S100 calcium-binding protein A9, a secreted protein associated with inflammation and poor survival in HCC patients, was considerably upregulated by TAMs, reinforcing stem cell-like features through the activation of NF-kB signaling.^342^ Furthermore, it has been demonstrated that TAMs may promote cancer stem cell maintenance by stimulating the TGF-β1/Smad2/3 pathway^343^ and the ERK1/2 pathway in glioblastoma.^344^ TAM-induced increase of CSC stemness in HCC^345^ and lymphoma^343^ may be attenuated by inhibiting the WNT/β-catenin pathway, as shown by a large body of in vivo and in vitro investigations. These findings prove that TAMs promote the formation, survival, and proliferation of CSCs and other stem cell subtypes (including mesenchymal stem cells) in TME.

### Reciprocal regulations of ageing and macrophages

The worldwide populace is aging, prompting an expanded future. Conversely, aging is related to falling apart health, an expanded danger of cancer, and diminished capacity of injury repair, putting massive weight on health foundations. Moreover, aging is linked to declining immune capacity. Therefore, the procedure is recognized as immunosenescence. Presentation to modified factors in an aging cell condition, ER stress, mitochondrial incapacity, distressed cellular digestion, and the innate immune variations in aging macrophages are mutually prone to assume an impact on phenotypic and functional alterations in macrophages.^346^ Likewise, age-related illnesses are linked with alterations to immune function, comprising the myeloid cells, and are associated with immunosenescence. Subsequently, the immunosenescence-linked age-associated changes correspond to immune dysfunction and low-grade chronic inflammation or inflammageing. Later, immune dysfunction is characterized by the rise in the expression of proinflammatory cytokines, including TNF, IL-16, and IL-1β, respectively.^347^

Immune signaling, enhanced cytokines, free fatty acids, hormones, immunoglobulins, and oxidized low-density lipoproteins all aggregate with aging and stimulate macrophages. Aging has appeared by specific investigations, yet not others, to conciliate recruitment of macrophage, phagocytosis, antigen presentation, cytokine generation, and ROS creation. Studies have demonstrated that murine macrophage phagocytosis stays unchanged at age.^348^ Peritoneal and splenic macrophages from older mice are less receptive to proinflammatory stimuli (LPS and IFN-γ) than younger mice. Generation of NO, TNF-α, IL-6, and ROS, subsequent presentation to LPS/IFN-γ in vitro diminish with the timespan of aging, demonstrating that encoding genes are inflammatory cytokines (IL-1β, IL-12, and IL-6), chemokines (CCL24) and their receptors (CCR3 and CCR5) were downregulated in splenic macrophages from normal and healthy old mice contrasted with their more vigorous and younger partners.^349^

Induction of MHC II is diminished on aging IFN-γ invigorated BM-determined macrophages contrasted with younger macrophages. Interestingly, aging peritoneal macrophages showed an improved generation of ROS and NO because of LPS. Macrophages in fat tissue and livers from old mice in vivo show an all the more professional inflammatory M1 phenotype than younger mice.^350^ Macrophages from aging lymphoid tissues yield expanded degrees of IL-10 with many Treg cells. Macrophages from elderly donors display diminished antigen-presenting limits contrasted with those from younger grown-up individuals.^351^ Modifications of exogenous incitements are also most likely to significantly help with the aging case in the inflammatory outline of macrophages. For instance, a decline in gut barrier function, as illustrated by reduced transepithelial electrical resistance, is predicted to be a notable cause for increased centralization of TLR ligands accessible for older adults. Likewise, other proinflammatory activators, such as advanced glycation end products and S100A8/A9, can increase significantly with advanced age in different tissues in the human body and mice, enacting macrophages by RAGE and TLR4.^352^

Baker et al.^353^ employed an inducible “senescence-to-apoptosis” progeric animal model, in which transgenic mice produce pro-apoptotic proteins under the regulation of the p16INK4a promoter, to determine whether senescent cells or apoptotic cells play a more significant role in tissue damage. Cells expressing the senescence-associated marker p16INK4a were turned into apoptotic cells in vivo after being given a “chemical switch” to the mice. Moreover, OCT4, SOX2, KLF4, and cMYC have recently been shown to cause cellular senescence and IL-6 production in vivo, resulting in more effective reprogramming.^354^ The newly discovered capacity of senescent cells draws similarities with macrophages in that both cells show metabolic markers like CD38 and produce substances that induce matrix remodeling and immunomodulation.^355^

In addition, senolytic drugs provide an approach to understanding better senescent cells and the macrophage’s precise depletion time. This study provides evidence that senolytics may stimulate SA-β-gal expression in cell culture. Senolytic drugs, such as ABT-737 or Dasatinib + Quercetin (DQ), when administered in vivo, promote apoptosis in senescent cells, leading to clearance in mice skin, lung, and hematopoietic tissues and so facilitating better tissue regeneration. Transplants from older mice have a better chance of surviving when DQ is given.^356^ Further, SASP may induce plasticity and stemness in somatic stem/progenitor cells, promoting a pro-regenerative response. Transient exposure to the SASP, as shown by Ritschka et al.,^357^ increases stem cell marker expression and regeneration potential in primary mouse keratinocytes in vivo. Senescence arrest was induced by SASP treatment and worked against the regeneration stimulus. And DQ, which targets BCL-2 family members and HIF-1α, PI3-kinase, and p21-related anti-apoptotic pathways, effectively reduces physical impairment in people with idiopathic pulmonary fibrosis (IPF). However, there are still constraints on the usage of senolytics, mainly owing to their lack of specificity, bioavailability, and method of administration.^358^

Evidence from animal models and human subjects indicates that the innate immune system, the body’s primary first line of defense, changes as we age. TLR1/2-induced TNF-α and IL-6 production are reduced in the blood monocytes of elderly adult patients, indicating a functional impairment in the monocyte/macrophage lineage. The ability of monocytes to phagocytose antigens is diminished with age, and the chemotaxis, MHC II expression, and antigen-presenting abilities of macrophages are also diminished.^359^ It has been shown that neutrophils lose their functionality as people age. Impaired immunological responses to vaccinations and infections and increased morbidity and mortality seen in senior populations are likely attributable to this age-associated malfunction of innate immune cells.^359^

Cleaning the body of senescent cells is another crucial role for macrophages. It has been hypothesized that the increase of senescent cells inside tissues leads to the malfunction and disease of organs that naturally occur with aging. In a progeroid environment (BubR1H/H mice), Baker et al.^353^ demonstrated that eliminating cells expressing the senescence-marker gene Cdkn2a (p16) delayed the development of age-related symptoms such as sarcopenia, cataracts, and adipose tissue loss.

## Immunotherapeutic advances targeting macrophages

There have been several immunotherapy approaches developed for cancer. These therapies include adoptive cellular immunotherapy, tumor vaccines, antibodies, immune checkpoint inhibitors (ICIs), and small-molecule inhibitors. Even if most of these tactics are not intended to target macrophages directly or were not designed initially, macrophages nonetheless have a substantial role in the ultimate results.

In addition to immune checkpoints on T cells, several checkpoints that are mostly connected with macrophages have also been found. Because of its association with SIRP on macrophages, CD47 is a poor prognostic marker in tumor cells, and this relationship helps tumor cells avoid phagocytic clearance by macrophages.^360,361^ Inhibition of tumor growth mediated by macrophages has been achieved by blocking CD47.^362^ Through its interaction with the beta-2 microglobulin (2M) portion of the MHC I complex, the inhibitory receptor LILRB1 that is found on macrophages stops tumor cells from being phagocytosed.^363^ By inhibiting macrophage phagocytosis, the CD24-Siglec-10 axis suppresses the immune system.^362^ The effectiveness of cancer immunotherapy has been dramatically improved by inhibiting these immunological checkpoints.

Vaccines used for therapeutic purposes are often developed to stimulate the production of protective T cells. Nonetheless, research conducted by Maxime Thoreau and colleagues showed that the benefits of a therapeutic vaccination might only be attained via the collaboration of T cells and macrophages. The combination of GM-CSF is used to boost the functions of DC and restrict the control of Tregs. Many therapeutic interventions employed GM-CSF as an adjuvant, such as Sipuleucel-T, STING agonist, and oncolytic virotherapy. These therapies were able to stimulate antitumor immune responses. However, GM-CSF has also been shown to activate macrophages to perform an anticancer role and induce M1 macrophage polarization. In a different study relating to the use of viruses in tumor immunotherapy, Wang and colleagues used an NF-κB-activating gene expression adeno-associated virus system to express an artificial neoantigen on the surface of tumor cells. This neoantigen could be targeted by particular immune cells. They found that macrophages could devour cancer cells when they used calreticulin, which is a signal that promotes phagocytic uptake.^364^ Exosomes that were produced from M1-polarized macrophages, as opposed to M2-polarized macrophages, were responsible for the enhancement of the anticancer vaccination. This was accomplished by inducing the production of Th1 cytokines and a more potent antigen-specific cytotoxic T-cell response.^365^ According to the findings of Xu and colleagues, a tumor vaccine based on listeria improved anti-PD-1 treatment against hepatocellular carcinoma by skewing the polarization of macrophages.^366^

Suppression of the recruitment of macrophages^398^ molecules on monocytes and macrophages, such as CCR2, CCR5, VEGFR, CSF1R, ITGA4, and C5a, contributes to the infiltration of macrophages into tumors; upregulation of inflammatory cytokines with inhibitors or antibodies directed against them or certain of their ligands (such as CCL2, CCL5, VEGF, and CSF1) could be able to prevent the recruitment of macrophages.^367^ Targeting Nrp1 and ANG2 may diminish angiogenesis, resulting in fewer macrophages being recruited to the site of the infection.^367^ Inhibitors of CSF1 are responsible for preventing the development of macrophages because CSF1 is an essential signal for the differentiation of macrophages. Apoptosis might be induced in macrophages with trabectedin, which could then be utilized to decrease their ability to survive. Immunotoxins that target the scavenger receptor-A or the folate receptor β (FRβ) have the potential to deplete TAMs, while bisphosphonates are metabolic analogs that diminish macrophages. It has been shown that an antibody that blocks Tim-3 can control the activation of TAMs. Anti-VEGF, anti-VEGFR, and tyrosine kinase inhibitors (TKIs) are three types of drugs that potentially impair the protumoral role of TAMs.^96,367^ These drugs work by decreasing angiogenesis. TAMs are responsible for an immunosuppressive microenvironment because they express indoleamine-pyrrole 2,3-dioxygenase (IDO), heme oxygenase, arginase, TGF-β, IL-10, prostaglandins, and a variety of other immunosuppressive molecules. Aspirin inhibits the production of prostaglandins in the body. It’s possible that relieving the function of other immune cells by blocking immune checkpoints (PD-L1, PD-L2, B7-H4, VISTA, B7–1, and B7–2) on macrophages may be beneficial.^368^ Interactions between CD47 on tumors and SIRP on macrophages, eliminating the macrophage blockade, assist tumor cells in evading phagocytosis.^369^ Antibodies directed against CD47 or SIRP could be able to clear the obstruction. The induction of repolarization of macrophages is a proven and effective method.^370,371^ TAMs polarized in the M1 state are linked to anticancer responses, while TAMs polarized in the M2 state are related to protumor activities. IFN, CD40 agonists, PI3Kγ/mTOR/DICER inhibitors, agonists of TLR4/7/8/9, methionine sulfoximine, HDAC inhibitors, and antibodies against macrophage receptors with collagenous structures are some of the stimuli that might cause M1 polarization. In contrast, substances that hinder M2 polarization, such as CSF1R inhibitors, corosolic acid, omeprazole, Gpr132 inhibitors, MEK/STAT3 inhibitors, fast-mimicking diets, and antibodies against IL-4, IL-4Rα, and IL-13, are also able to diminish the amount of tumor burden.^372^

### Cancer therapy, immunomodulation, and therapeutic resistance

Patients with potentially curable cancers undergo two phases of systemic chemotherapy: neoadjuvant treatment before surgery and adjuvant treatment afterward. Adjuvant therapy is given to patients following surgery to raise their chances of survival and lengthen the time they are free of diseases (metastases-free).^373^ With neoadjuvant chemotherapy (NAC), patients may undergo curative-intent surgery after having a smaller primary tumor and less widespread regional lymphadenopathy.

After chemotherapy, TAMs tend to congregate in tumors. They might promote tumor recurrence by firing off the physiological regeneration program, which is helpful for wound healing but counterproductive regarding tumor relapse.^374^ All interconnected processes are macrophage-induced inhibition of T-cell immunity, tumor cell survival preservation, and tumor revascularization stimulation.

Increased recruitment of immunosuppressive TAMs, pro-tumor polarization, decreased T-cell cytotoxic response, and activation of anti-apoptotic programs in malignant cells are all mechanisms responsible for the tumor-promoting activity of TAMs after chemotherapy and chemoresistance.^375^ a. TAMs that promote doxorubicin resistance display high levels of CD68, CD206, CD163, and PD-L1 but low levels of CD80 and CD86. In addition, TAMs secrete pro-angiogenic VEGF, which causes revascularization, and immunosuppressive cytokines (IL-10, TGF). PD-L1 overexpression in TAMs suppresses the antitumor capabilities of cytotoxic T cells, allowing tumor cells to survive, proliferate, and develop chemoresistance. b. STAT3 signaling and macrophage-produced IL-6 that promotes tumor cell proliferation are linked to TAM-mediated resistance to carboplatin. By activating the PI3K/AKT signaling pathway, TAM exosomes contribute to tumor cell cisplatin resistance. By inhibiting caspase-3 activation and death in tumor cells, chemoresistance to gemcitabine is mediated. c. Cathepsin B and S, proteases released by TAMs, induce chemoprotection directly through NF-κB activation or indirectly via IL-6 production and STAT3 activation. The precise ways in which chemotherapy affects macrophages remain unclear.^375^

As an alternative, activated M2 macrophages may mediate chemoresistance by shielding tumor cells from chemotherapy’s harmful effects via the secretion of growth factors and inhibiting cell death signaling pathways.^376^ The infiltration of CD68^+^ and CD163^+^ macrophages in tumor mass is strongly correlated with tumor depth, lymphatic and venous invasion, and poor prognosis in patients with esophageal cancer who underwent neoadjuvant chemotherapy (two cycles of 5-fluorouracil (5-FU), cisplatin, and adriamycin).^377^ After chemotherapy-induced tumor revascularization and relapse in a mouse Lewis lung carcinoma model (LLC1s) and mouse models of breast cancer metastasis (MMTV-PyMT), a substantial increase in CD206^+^ TAMs was observed, with these cells accumulating primarily in the vascularized chemokine CXCL12-rich regions of tumors.^374^ A significant rise in macrophages protecting tumors was seen in breast cancer patients following neoadjuvant chemotherapy and in the PyMT mice model after paclitaxel (PTX) treatment.^378^ Using chemotherapeutic drugs, such as the DNA-damaging chemical trabectedin, which has a potent anticancer effect, may also harm monocytes and macrophages.^367^ Treatment with trabectedin dramatically slowed tumor development and reduced production of the primary monocyte chemoattractant CCL2 by TAMs in transplantable tumor models of fibrosarcoma, ovarian carcinoma, and Lewis lung carcinoma. One proposed mechanism for trabectedin’s anticancer action is lowering CCL2 levels, reducing the number of macrophages in tumor tissues.^379^

The possibility of using chemotherapy to set up immune responses that kill tumor cells was also examined. Therefore, anticancer medicines that alter cellular DNA cause tumor cells to display neoantigens, leading to immunogenic cell death (ICD). To put it simply, carboplatin is a platinum compound that contains DNA-damaging chemicals.^380^ Patients with stage II-III triple-negative breast cancer (TNBC) who were given carboplatin had a higher rate of tumor pathological complete response (pCR) than those given a placebo (36% vs. 23.8%).^381^ In the BrighTNess study, the pCR rate in TNBC rose from 31% without carboplatin to 58% with carboplatin.^381^ Patients with gastric cancer who underwent postoperative chemotherapy based on 5-fluorouracil (FU) lived longer if their pretreatment TAM levels were high.^382^ In patients with stage III CRC treated with 5-FU adjuvant therapy, the high macrophage density before the treatment was strongly linked with a better-improved prognosis.^383^ Increased numbers of CD68^+^ TAMs in patients with pancreatic adenocarcinoma before treatment were only related to a favorable prognosis in those who received adjuvant gemcitabine-based chemotherapy but not in those who did not. Due to a substantial increase in cellular ROS generation, gemcitabine (GEM) re-educated macrophages to an anticancer phenotype in vitro.^384^ In the GEM-modified polarization of macrophages, the expression of M1 markers HLA-DR, CD40, and the chemokine receptor CCR7 was upregulated, whereas the expression of M2 markers CD163 and CD206 was downregulated, and the pro-inflammatory program was activated.^384^

Thus, TAM’s function in tumor growth and chemoresistance in cancer is highly debated. The direction of these changes (enhancement or reduction) depends on the kind of CT agent and the type of cancer. Macrophages influence the action of chemotherapy medications. Mechanistically understanding how various TAMs react to CT agents is essential. Much research has been done on the function of TAMs in radiotherapy and chemotherapy. Two frequently used cancer treatments, and TAMs have been linked to a decline in cancer chemotherapy’s effectiveness. Ultimately, chemotherapeutic drug resistance^385^ resulted from CCL5 produced by TAMs, which activated STAT3 and caused Nanog overexpression. After docetaxel and androgen deprivation treatment, prostate cancer cells secreted more CXCL12 via TAMs,^386^ which helped cancer cells survive and reduced their sensitivity to chemotherapy by activating CXCR4. When treating advanced NSCLC, EGFR-TKIs are a novel approach.^387^

Patients with progressive illness after EGFR-TKI therapy had greater TAM levels than those without advanced disease, according to a study by Chung et al. that analyzed 206 instances of NSCLC patients. As a result, increased TAM counts were substantially correlated with shorter progression-free survival and overall survival, which suggests that TAMs are connected to decreased treatment responsiveness following EGFR-TKI administration.^388^

TAMs, on the other hand, could improve radiotherapy’s efficacy. After radiation, TAMs are more likely to be recruited into tumors, where they may influence how tumor cells respond to therapy.^389^ It was shown by Stafford et al.^390^ that the CSF-1R inhibitor PLX3397 might suppress the differentiation of myeloid monocytes into TAMs, hence enhancing the responsiveness of glioblastoma to ionizing radiation therapy and postponing recurrence. PM37 inhibited TAM-induced protein kinase C zeta and IL-4/IL-13-mediated STAT6 tyrosine phosphorylation, decreasing TAM-mediated radioresistance of inflammatory breast cancer cells.^391^

Furthermore, additional investigations showed that inhibiting TAM or TAM-related signaling pathways enhanced the effectiveness of radiotherapy.^392,393^ In short, TAMs are a double-edged sword since they may both promote tumor clearance and improve cancer growth and therapeutic tolerance. Thus, further research is needed to determine their roles in carcinogenesis.

### Mechanisms in tissue repair

Although many tissue’s resident tissue macrophage population originates in the yolk sac and fetal liver during development, they are supplemented by inflammatory monocytes drawn from the bone marrow in response to tissue damage.^394^ DAMPs, PAMPs, growth factors, cytokines, and other mediators generated in the local tissue microenvironment cause dramatic phenotypic and functional changes in the recruited and resident macrophages. Many distinct substances are secreted by macrophages that promote the growth, differentiation, and activation of cells, such as fibroblasts, epithelial cells, endothelial cells, and stem and progenitor cells, all essential for tissue repair. By the time the repair process is nearing completion, these cells have taken on a regulatory pro-resolving phenotype, guaranteeing that the inflammatory response that damages tissue is dampened and typical tissue architecture is restored. Fibrosis, the damaging scarring of tissues, may result from unchecked inflammation and/or maladaptive healing mechanisms. Even while recruited monocytes sometimes seed tissues and take on a resident macrophage phenotype, the processes that ultimately lead to a return to tissue homeostasis remain unclear.^394^

Most macrophages adopt a wound-healing phenotype once the inflammatory phase has subsided. This phenotype is defined by synthesizing growth factors such as PDGF, TGF-β1, IGF-1, and VEGF-α, stimulating cellular proliferation and blood vessel creation.^395^ Over time, macrophages with a predominantly anti-inflammatory phenotype will become the norm. These macrophages can respond to IL-10 and other inhibitory mediators and secrete anti-inflammatory mediators such as IL-10 and TGF-β. Cell surfaces express receptors such as PD-L1, and PD-L2 play crucial roles in suppressing the immune system and silencing the inflammation that, if not controlled effectively, can lead to collateral cell death and ultimately delay the repair process. Recent research points to a population of CD11b^+^ macrophages that regulates the damage and recovery stages of tissue healing. Another study shows that macrophages in the heart are produced from CCR2^+^ monocytes and drive the first inflammatory response after tissue damage.^394^

Recent research suggests that macrophages activated by type-2 cytokines can exhibit potent anti-fibrotic activity, especially when the tissue repair response becomes chronic.^396,397^ This is because they can antagonize the function of proinflammatory M (IFN-γ) macrophages, which exacerbate tissue damage. Mechanistic investigations of M(IL-4)-skewed macrophages in chronic models of fibrosis and cancer support the idea that these cells decrease local CD4^+^ Tcell responses and reduce ECM formation by myofibroblasts, hence slowing fibrosis development and promoting cancer growth and metastasis.^398^ M(IL-4)-skewed macrophages, which are found in tumors and granulomas, are closely associated with other inflammatory cells and actively compete with neighboring T cells and myofibroblasts for the amino acids L-arginine and L-ornithine, which become depleted in areas of hypoxia but are crucial for the maintenance of local T cell proliferation and myofibroblast activation.^394^

### Macrophages-targeted therapy

Over the past few decades, substantial preclinical and clinical progress has been made in understanding macrophage biology and its clinical relevance in human diseases. Therefore, macrophage-targeted therapy is emerging, and some have been translated into clinical trials.^367,399^ Several immunotherapeutic approaches may benefit from macrophage depletion, such as CCL2 vaccination [406] and ICIs such as PD-1 and CTLA4 [407–409]. Anti-CSF1R antibodies and other treatment methods focused on TAMs are now being tested in many ongoing clinical studies (Table 3).^282,310,367^ Moreover, over the last several years, macrophages have gained more and more attention as a potential immunotherapy component for treating cancer. Due to their usefulness in existing therapeutic approaches, they have emerged as a prime candidate for future advances in cancer therapy. Immunotherapy has emerged as the gold standard, given the shortcomings and shortages of conventional cancer therapies. Several FDA-approved cancer immunotherapy therapies use direct and indirect macrophage targeting (Table 4).

**Table 3:** Therapeutic targets in clinical trials targeting macrophages in cancers

Target/drug	Sponsor/organization/agent	Clinical Identifier/intervention	Type of cancer	Clinical Trial
CCL2	Centocor Research & Development, (Carlumab (CNTO 888)	NCT00992186	Prostate cancer	Phase II
CSF1	Novartis Oncology, (Lacnotuzumab (MCS110)	NCT02435680; (Carboplatin, gemcitabine)	Advanced triple-negative cancer	
		NCT01643850	Pigmented villonodular synovitis	
		NCT03694977; (PDR001)	Gastric cancer	
TIE2	Karmanos Cancer Institute, (CEP-11981 (ESK981)	NCT04159896; (Nivolumab)	Prostate cancer	
		NCT03456804	Prostate cancer	
TIE2	Bayer, (Regorafenib (BAY 73–4506)	NCT04170556 (Nivolumab); NCT04476329	Hepatocellular carcinoma	
Arginase	Incyte, (INCB001158 (CB1158)	NCT02903914; (Pembrolizumab); NCT03314935; (Oxaliplatin, leucovorin, 5-fluorouracil, gemcitabine, cisplatin, paclitaxel); NCT03837509 (Daratumumab)	Advanced/metastatic solid tumors; Solid tumors; Multiple myeloma	
CD40	Celldex Therapeutics, (CDX-1140)	NCT04491084; (CDX-301); NCT04364230	Non-small-cell lung cancer, lung cancer; Melanoma;	
	Genentech, Inc.Seagen Inc., (Dacetuzumab (SGN-40)	NCT00283101	Lymphocytic, chronic leukemia	
		NCT00435916	Large B-cell diffuse lymphoma, non-Hodgkin lymphoma	
	Novartis Pharmaceuticals, (Lucatumumab (HCD122)	NCT00670592	Non-Hodgkin’s lymphoma, Hodgkin’s lymphoma	
BTK	Pharmacyclics LLC, (Ibrutinib (PCI-32765)	NCT02599324	Renal cell, urothelial, gastric, colon, pancreatic adenocarcinoma	Phase II
		NCT01752426; (heavy water (2H_2_O)	Leukemia	
		NCT01236391	Mantle cell lymphoma	
		NCT01105247;	B-cell chronic lymphocytic leukemia, small lymphocytic lymphoma	
		NCT01614821;	Waldenstrom’s macroglobulinemia	
		NCT01520519; (Rituximab)	Leukemia	
		NCT01109069;	B-cell lymphoma, chronic lymphocytic leukemia	
		NCT01217749; (Ofatumumab)	Chronic lymphocytic leukemia	
		NCT02403271; (Durvalumab)	Non-small-cell lung cancer, breast cancer, pancreatic cancer	
		NCT01646021; (Temsirolimus)	Mantle cell lymphoma	Phase III
		NCT01855750; (Rituximab, cyclophosphamide, doxorubicin, vincristine, prednisone)	Lymphoma	Phase II
	Pharmacyclics LLC	NCT01980628;	Marginal zone lymphoma, B-cell lymphoma	
		NCT01589302;	Prolymphocytic leukemia, small lymphocytic lymphoma, chronic lymphocytic leukemia	
		NCT01325701;	Diffuse large cell B lymphoma	
		NCT01578707; (Ofatumumab)	Chronic lymphocytic leukemia, small lymphocytic lymphoma	Phase III
		NCT01722487; (Chlorambucil)	Chronic lymphocytic leukemia, small lymphocytic lymphoma	
		NCT0243666;8 (Gemcitabine, nab-paclitaxel)	Metastatic pancreatic adenocarcinoma	
		NCT01980654; (Rituximab)	Follicular lymphoma, B-cell lymphoma, non-Hodgkin’s lymphoma	Phase II
		NCT01973387; (Rituximab)	Chronic lymphocytic leukemia, small lymphocytic lymphoma	Phase III
		NCT01611090; (Bendamustine, hydrochloride, rituximab)	Chronic lymphocytic leukemia, small lymphocytic lymphoma	
		NCT02401048; (MEDI4736)	Diffuse large B-cell lymphoma, follicular lymphoma	Phase II
		NCT02639910; (Tafasitamab, idelalisi, venetoclax)	Chronic lymphocytic leukemia, small lymphocytic lymphoma	
		NCT02902965; (Bortezomib dexamethasone)	Multiple myeloma	
		NCT01744691	Chronic lymphocytic leukemia with 17p deletion, small lymphocytic lymphoma with 17p deletion	
		NCT02264574; (Obinutuzumab, chlorambucil)	Chronic lymphocytic leukemia, small-cell lymphoma	Phase III
		NCT02514083; (Fludarabine)	Chronic lymphocytic leukemia, small lymphocytic lymphoma	Phase II
	Acerta Pharma BV, (Acalabrutinib (ACP-196)	NCT02180724	Waldenström macroglobulinemia	
		NCT02213926	Mantle cell lymphoma	
	BeiGene, (Zanubrutinib (BGB-3111)	NCT03206970	Mantle cell lymphoma	
		NCT03206918	Chronic lymphocytic leukemia, small lymphocytic lymphoma	
CSF1R	Plexxicon, (Pexidartinib (PLX-3397)	NCT01596751; (Eribulin)	Metastatic breast cancer	Phase II
	Array Biopharma, (ARRY-382)	NCT02880371; (Pembrolizumab)	Advanced solid tumors	
	Eli Lilly (IMC-CS4(LY3022855)	NCT03101254; (Vemurafenib cobimetinib)	Melanoma	
	Five Prime Therapeutics (Cabiralizumab (FPA008))	NCT02471716	Tenosynovial giant cell tumor	
		NCT03927105	Peripheral T-cell lymphoma	
		NCT04331067; (Nivolumab)	Triple-negative breast cancer	
	Hoffman La Roche (Emactuzumab (RO5509554))	NCT03708224 (Atezolizumab)	Advanced head and neck squamous cell carcinoma	
		NCT03193190; (Additional therapies)	Pancreatic ductal adenocarcinoma	
	Deciphera Pharmaceuticals LLC (DCC-3014)	NCT03069469	Advanced malignant neoplasm	
	Syndax (SNDX-6532)	NCT04301778; (Durvalumab)	Unresectable intrahepatic cholangiocarcinoma	
	Plexxikon (PLX3397)	NCT02584647; (Sirolimus)	Sarcoma, nerve-sheath tumors	
		NCT02452424; (Pembrolizumab)	Advanced melanoma and solid tumours	
	Novartis (BLZ945)	NCT02829723PDR001 (anti-PD1)		
CD47	Gilead Sciences (Magrolimab (Hu5F9-G4))	NCT04541017 (Mogamulizumab)	T-cell lymphoma	
		NCT04435691 (Azacitidine, venetoclax)	Acute myeloid leukemia	
		NCT03869190 (Atezolizumab, enfortumab, vedotin, niraparib)	Urothelial carcinoma	
		NCT02953509 (Rituximab, gemcitabine, oxaliplatin)	Non-Hodgkin lymphoma	
		NCT04313881 (Azacitidine)	Myelodysplastic syndromes	Phase III
	Arch Oncology (AO-176)	NCT03834948 (Paclitaxel)	Solid tumor	Phase II
		NCT04445701 (Bortezomib, dexamethasone)	Multiple myeloma	
CCR2	Bristol-Myers Squibb (BMS-813160)	NCT03184870 (Chemotherapy or nivolumab)	Colorectal/pancreatic cancer	
		NCT03496662 (Nivolumab abraxane, gemcitabine)	Pancreatic cancer	
		NCT03767582 (Radiation therapy, nivolumab, GVAX)	Pancreatic cancer	
		NCT04123379 (Nivolumab, BMS-986253)	Non-small-cell lung cancer, hepatocellular carcinoma	
		NCT02996110 (Nivolumab, ipilimumab, relatlimab, BMS-986205)	Advanced cancer	
	ChemoCentryx (CCX872-B)	NCT03778879 (Radiation therapy)	Pancreatic cancer	
	Millenium (MLN1202)	NCT01015560	Bone metastases	
	Pfizer (PF-04136309)	NCT02732938 (Nab-paclitaxel, Gemcitabine)	Metastatic pancreatic ductal adenocarcinoma

**Table 4:** FDA-approved drugs targeting macrophage in cancers

Drug	Sponsor/company	Mechanism of action/administration	Type of cancer	FDA approval	Ref.
Plexxikon, (Pexidartinib (PLX-3397)	Daiichi Sankyo	Orally administered Pexidartinib is a small-molecule TKI with robust and specific action against the CSF1 receptor. CSF1, is overexpressed in many solid tumors, promotes the survival of TAMs and the development of monocytes into TAMs; capsules	Tenosynovial giant cell tumor	2019	^441^
Trabectedin (Yondelis)	Janssen	Induces apoptosis through the tumor necrosis factor receptor superfamily member 10 (TNFRSF10, also known as TRAIL) in monocytes and TAMs, reducing their numbers in human patients and mice; intravenous infusion.	Soft tissue sarcomas, ovarian cancer	2015	^379^
Sipuleucel-T (Provenge)	Dendreon	It is a fusion protein consisting of GM-CSF and prostatic acid phosphatase, which is utilized to stimulate antigen-specific T lymphocytes against the tumor.	Prostate cancer	2010	^59^
Carboplatin	Bristol-Myers Squibb	Treatment with platinum-based neoadjuvant chemotherapy has been shown to decrease markers associated with alternative macrophage activation. Macrophage depletion by CSF1R inhibitors (CSF1Ri) in the mouse models provides further evidence of a switch in TAM functions; intravenous infusion	High-grade ovarian cancer	2003	^442^
Paclitaxel (Taxol)	Mylan Pharmaceuticals			2002	
5-fluorouracil (Eloxatin)	Sanofi	The synergistic impact between 5-fluorouracil and macrophages leads to enhanced CRC cell death; intravenous infusion	Colorectal cancer	Initial approval in 1962, 2002 for CRC	^383^
Gemcitabine	Eli Lilly	Changes in innate immune cells, including increased infiltration of protumoral M2 TAMs and metabolic reprogramming, are an outcome of gemcitabine treatment; intravenous infusion	Pancreatic ductal adenocarcinoma (PDAC)	1996	^384^

As one of the promising treatments, immunotherapy has dramatically reshaped the landscape of tumors with exceptional clinical outcomes. However, only a minority of patients respond to ICIs, cancer vaccines, and infusing cell-based therapies. Evidence indicates macrophage-targeted immunotherapy potently enhances adaptive protective immunity against tumor growth, progression, and metastasis.^367^ With single-cell transcriptomic data and generation sequencing, researchers focus on understanding the complexity and diversity of macrophages with different biomarkers, macrophage states for disease progression, mechanistic studies of TAM functions, and rational manipulation of macrophages as an effective anti-tumor strategy.^393^ According to clinicaltrials.gov, 1759 clinical trials with macrophage-associated clinical trials were registered in 2022. There are more than 554 clinical trials with macrophage-based cancer therapies (search terms ‘macrophage’ or ‘macrophage’ with ‘cancer’).

In cancers, an effective scheme is the depletion of TAMs in the TME to counter their negative impact. Bisphosphonates can be taken up by phagocytes to deplete TAMs by inducing cell apoptosis. Currently, bisphosphonates are used clinically with decreased disease recurrence, metastasis, and overall mortality for breast cancer.^400^ Among them, clodronate, one of the non-nitrogen bisphosphonates, is artificially loaded by liposomes. It can induce apoptosis of macrophages and inhibit tumor growth.^401^ Zoledronate, a third-generation nitrogen-containing bisphosphonate, has been shown to exhibit selective cytotoxicity towards MMP9-expressing TAMs and reduce the infiltration of TAMs, decrease tumor angiogenesis, and inhibit tumor progression.^402^ Similarly, BLZ-945 (a CSF-1R inhibitor) and chemotherapy drugs (such as doxorubicin and epirubicin) can specifically target and deplete TAMs.^403^ In addition, inhibiting macrophage recruitment is the second strategy for TAM-targeting strategy treatment. Many inhibitors, such as inhibitors of ANG2 (Trebananib), CCL2/CCR2 (Carlumab and PF-04136309), CCL5/CCR5 (Leronlimab, Maraviroc, and Maraviroc), CSF-1/CSF-1R (Emactuzumab and Pexidartinib), and VEGF have been shown to inhibit macrophage recruitment for tumor growth.^404^

Macrophage reprogramming is crucial to reshaping their potential immune-stimulatory role as the significant phagocytes and professional antigen-presenting cells (APCs) within the TME. Generally, normal cells can express anti-phagocytosis molecules called “phagocytosis checkpoints” to avoid self-elimination by phagocytes. Signal regulatory protein alpha (SIRPα) is a vital immunoreceptor tyrosine-based inhibitory motifs (ITIM)-bearing inhibitory receptors expressed on macrophages. Tumor cells can become active in a “don’t eat me” signal and avoid macrophage phagocytosis by over-expression of CD47 to recognize SIRPα, thereby leading to patients’ poor survival.^405^ Studies showed that blocking the CD47-SIRPα interaction by CD47 antibodies, a phagocytosis checkpoint inhibitor promotes phagocytosis in TAMs and enhances cancer immunotherapy, chemotherapy, and other combined therapy.^406^ Garcia et al. reported that the combination of anti-CD47 antibody and PD-L1 blockade improved innate and adaptive immune checkpoint response rates and potentiated the vaccinal effect of antitumor antibody therapy in a mouse B16F10 model.^407^

Reprograming M2-like TAMs toward M1-like TAMs represents an attractive strategy for macrophage-targeting treatment. CSF1/CSF1R signaling pathway has positive roles in macrophage biology, including survival, proliferation, differentiation, and phagocytosis.^408^ Stephen et al. reported that CSF-1R blockade with PLX3397 improved the efficacy of adoptive cell therapy (ACT) in the mouse melanoma model.^409^ CSF-1R blockade reduced the ability to unleash the immune-stimulatory capacity of TAMs with a skewing of MHC II^low^ to MHC II^hi^ macrophages. In addition, macrophage treatment with CD40 agonists, such as Sotigalimab and Selicrelumab, can significantly upregulate the expression of MHC, promotes the secretion of inflammatory cytokines, actives DCs, and induce cell polarization of M1-like TAMs.^310^ Furthermore, in clinical trials, blocking PI3Kγ by Eganelisib or Umbralisib has been developed to turn on an “immune-stimulatory program” in immunosuppressive macrophages. This dramatic shift of TAMs is benefit in modulating the TME and promoting ICIs treatment against cancers.^410^ Many macrophage-targeting agents have been developed with different approaches for cancer therapy, including previously unmentioned CXCL12-CXCR4 inhibitors, TREM inhibitors, SIGLEC10-CD24 inhibitors, and TLR agonists.^404^

In other studies, Chen et al. demonstrate that intratumoral high potassium inhibits the anti-tumor capacity of TAMs via Kir2.1, which supports that genetic depletion or pharmacological blockade of Kir2.1 repolarizes TAMs toward the antitumor state.^132^ In addition, TAM-targeted CAR T cells are a novel tool to eliminate TAMs and promote antitumor functions of TAMs, thereby modifying the TME and inducing anticancer immune responses.^404,411^ For example, folate receptor-β-targeted CAR T cells can eliminate human M2-like macrophages and mouse M2-like TAMs and promote cell proliferation of M1-like cells and tumor-specific T cells in TME.^411^ Lastly, engineered macrophages, nanoparticle-assisted drugs, and oncolytic viruses have been proven superior candidates for cancer macrophage-based immunotherapy and drug delivery.^404,412–416^

The host’s immune system triggers autoimmune diseases and inflammation to self-antigens, damaging the normal tissues. RA and inflammatory bowel disease (IBD) are mainly induced by prolonged inflammation. Feng et al.^417^ pointed out that small interfering RNA (siRNA) nano drugs targeting the endoplasmic reticulum to nucleus signaling 1 (ERN1) gene (siERN1) can mediate macrophage polarization and have significant therapeutic effects in mouse collagen-induced arthritis and inflammatory bowel disease models. In addition, macrophages are all-rounders in kidney injury repair and kidney fibrosis. Studies have shown that reprogramming the metabolism of macrophages is a promising target treatment for kidney dysfunction. To date, no effective acute kidney injury (AKI) treatments have been implemented. Satoko et al.^418^ showed that the apoptosis inhibitor of macrophage protein on intraluminal debris could interact with kidney injury molecule (KIM)−1 and ameliorated renal pathology, thereby promoting recovery from AKI. Interestingly, extracellular vesicles (EVs), including exosomes from macrophages, have been shown to control inter-cellular communication in numerous disease states, including inflammation and metabolic disease. Thus, EVs-based treatments attracted extensive concern for anti-inflammation. Phu et al.^419^ reported that IL-4 polarized human macrophage exosomes significantly control cardiometabolic inflammation and diabetes in obesity. KCs, a kind of macrophage in the liver, play a central role in nonalcoholic steatohepatitis (NASH) etiology. KCs produce endogenous miR-690 via exosome secretion to directly inhibit fibrogenesis in HSCs, inflammation in recruited hepatic macrophages (RHMs), and de novo lipogenesis in hepatocytes. These studies suggest that miR-690 in exosomes could emerge as a therapeutic for NASH.^420^ The coronavirus disease 2019 (COVID-19) pandemic has placed an excessive burden on human health, with hypercytokinemia and inflammation. Timothy et al.^421^ reported that baricitinib, a clinically approved JAK1/JAK2 inhibitor, can exploit as a frontline drug for inflammation induced by SARS-CoV-2 infection. Rhesus macaques treated with baricitinib showed a rapid and remarkably potent suppression of lung macrophages with lower production of cytokines and chemokines, decreased lung infiltration of inflammatory cells, reduced NETosis activity, and more limited lung pathology. Thus, out-of-balance of the yin and yang of macrophages is a double-edged sword and performs different functions in cancers, autoimmune diseases, and disease inflammation. Strategies of macrophage-targeted therapy may vary in certain diseases.

## Conclusions and perspectives

Macrophages display substantial heterogeneity in immune function and phenotypes that might alter into diverse subtypes of cells in response to contact with numerous immune microenvironments. These variations, also known as macrophage polarization, play a significant role in inflammation and disease and are vital for instigation, differentiation, and survival. Lack of macrophages or differentiation injury in the aforementioned immune cells is the foundation for the pathogenesis of many infections and disorders. Metabolic, mitochondrial, and transcriptions alterations might be the core reason for this pivotal loss of immune cells due to transformed cytokine production, transcription, and changes in immune pathways. Also, pathogen or inflammatory signals trigger macrophage differentiation to procure novel roles by swiftly modulating immunity by critical gene expression.

Autophagy is a critical macrophage function that is crucial for pathogenic host defense. Furthermore, it degrades advancing cellular pathogens by lysosomes and regulates the inflammatory responses to confine the host damage.^422^ As a vital member of the TME subverting immune cells, macrophages lead to the growth and development of clinically significant cancers. The regulatory mechanisms of macrophage polarization are critical for TME.

In addition to other functions of macrophages, aging is a multifaceted strategy that fundamentally impacts all organs. Deteriorated cellular repair roots augmented injury at genomic and proteomic stages upon aging. Tissue macrophages are central inflammatory cytokine fabricators, additional stimulators, and regulators of aging inflammation. Repair damage may contribute to systemic changes in metabolism and the production of pro-inflammatory cytokines, resulting in lower-grade inflammation or ‘inflammation’.^352^ Comprehending the interaction between macrophage and immune regulation approaches and tumorigenesis is central to gaining precise functions. It is also evident that the numerous immune signaling mechanisms are robustly intertwined, and feedback loops play a role in intensifying or inhibiting immune responses.

Therefore, as rising literature describes the progressions and immune pathways in macrophages, the eventual objective is a unified interpretation of detailed mechanisms of macrophages that are chief triggering factors, effectors, and advanced immune regulators of numerous inflammatory responses.

The diversity and unique properties of TAMs identified in tumors provide the groundwork for creating tailored therapeutic approaches that are TMA-based. In light of the multifaceted part that TAMs play in the process of tumor formation, we need more in-depth research on the roles of TAMs and the regulatory mechanisms governing them to find effective anti-tumor targets. Targeting TAMs can potentially result in the reversal of the TME concerning tumor promotion and immune suppression. This makes TAM targeting a potentially innovative therapeutic strategy for future precision cancer therapies. Many questions still have not been solved concerning the essential molecules or signals responsible for the functional reprogramming of TAMs. To find the answers to these issues, you will need to have a deeper grasp of the regulation of TAMs. In conclusion, TAMs have a wide range of functions and perform a variety of essential roles in the TME. It is possible that targeted therapy of TAMs, either alone or in conjunction with more traditional therapeutic techniques, may give additional insight into potential future treatment options for cancer.
